# An Enhanced Hybrid SVD-DBO-VMD Framework for Rolling Bearing Fault Feature Extraction Using Vibration Data

**DOI:** 10.3390/s26175485

**Published:** 2026-08-29

**Authors:** Chen Zhang, Luyan Xu, Xiansong He, Zhibin Zhao, Xiaoli Zhao

**Affiliations:** 1 Department of Armored Vehicle Technology Campus of Urumqi, Engineering University of PAP, Urumqi 830049, China; jxkpzx01@163.com; 2 School of Mechanical Engineering, Nanjing University of Science and Technology, Nanjing 210094, China; brownsong@njust.edu.cn; 3 School of Mechanical Engineering, Xi’an Jiaotong University, Xi’an 710049, China; zhaozhibin@xjtu.edu.cn

**Keywords:** VMD, SVD, DBO, feature extraction

## Abstract

To address the challenging problem of extracting fault information from rolling bearings, this study proposes a novel hybrid method that integrates singular value decomposition (SVD), Dung Beetle Optimization (DBO), and Variational Mode Decomposition (VMD) for enhanced fault feature extraction. The method consists of three key steps: (1) adaptive SVD denoising via singular-value difference spectrum, where the collected signal is first reconstructed into a phase-space Hankel matrix, and the maximum extremum point of its singular value difference spectrum is identified as the optimal rank order for SVD denoising; (2) DBO of VMD parameters using minimum envelope entropy—to prevent over-decomposition, where DBO is employed to automatically determine the optimal number of modes K by minimizing the envelope entropy, and the signal is decomposed into a set of Intrinsic Mode Functions (IMFs); (3) kurtosis-peak-to-peak joint screening of sensitive IMFs, in which a joint criterion based on kurtosis and peak-to-peak values is applied to select the IMF that contains the richest fault information. This sensitive IMF is then subjected to Hilbert envelope spectrum analysis to accurately extract the fault characteristic frequency of rolling bearings. Experimental results from two different test rigs demonstrate that the proposed method can more effectively highlight periodic fault impulses and identify fault types, offering a reliable approach for rolling bearing fault feature extraction.

## 1. Introduction

Rolling bearings are critical components in rotating machinery, yet they are prone to damage. During operation, the damaged elements of the bearing repeatedly contact other components, generating periodic impact signals. In theory, these periodic impacts and the time intervals between adjacent impacts are key features for identifying local damage faults. However, in practical engineering applications, factors such as complex vibration transmission paths and severe environmental noise interference make the early identification of bearing faults particularly challenging. Effective analysis and utilization of rolling bearing vibration signals to extract impact characteristics and identify impact frequencies are crucial for the fault diagnosis of rolling bearings.

In recent years, numerous scholars have conducted extensive research on the early fault diagnosis of rolling bearings. Studies indicate that early fault diagnosis of bearings typically involves three steps, data acquisition, fault feature extraction, and fault frequency detection, with fault feature extraction receiving widespread attention [1,2]. Current fault feature extraction methods based on vibration signals include filtering methods, wavelet transform, empirical wavelet transform, empirical mode decomposition, ensemble empirical mode decomposition, local mean decomposition, Variational Mode Decomposition, sparse decomposition, and singular value decomposition, among others. Li et al. [3] proposed a bearing fault diagnosis method based on spectral kurtosis-optimized adaptive bandpass filtering and Hilbert envelope demodulation, which can effectively determine the optimal frequency band for fault diagnosis. However, the performance of the bandpass filtering method is influenced by the center frequency and bandwidth. Guo et al. [4] applied multi-scale wavelet decomposition and feature fusion for rotating machinery fault diagnosis, which is effectively used for bearing fault detection. Nevertheless, the application of wavelet transform in fault diagnosis depends on the selection of the wavelet basis. Gao et al. [5] introduced the adaptive generalized empirical wavelet transform (EWT) for rolling bearing fault diagnosis. However, EWT can cause issues such as excessive spectral division and unreasonable division in spectral segmentation. When applying the empirical mode decomposition (EMD) method combined with genetic algorithm and back propagation neural network for rolling bearing health status evaluation, this decomposes the signal into the sum of several Intrinsic Mode Functions (IMFs) according to the local characteristic time scales of the signal. However, the EMD method suffers from problems such as endpoint effect and mode mixing, and its performance is affected by the iteration criteria [6]. To overcome the shortcomings of EMD, Wang et al. [7] proposed an adaptive complete ensemble empirical mode decomposition with adaptive noise (CEEMDAN) method for rolling bearing fault diagnosis, which utilizes the statistical properties of white noise. However, the performance of the CEEMDAN method is influenced by the number of ensembles. Kumar [8] discussed the local mean decomposition (LMD) method in the context of non-stationary condition monitoring, noting that LMD still exhibits endpoint effects and other phenomena. Peng et al. [9] proposed a cyclic band Box-Cox sparse measures based blind filtering method for extracting bearing fault features, which has been successfully applied to bearing fault diagnosis. However, the performance of the sparse decomposition method is affected by main parameters such as the quality factor.

Singular value decomposition (SVD) and Variational Mode Decomposition (VMD), as effective nonlinear filtering methods, are widely used in mechanical fault diagnosis [10,11,12,13,14,15,16,17]. SVD is primarily used in signal processing for the extraction of periodic components and noise reduction, and it exhibits excellent invariance and stability. By utilizing the maximum protrusion point of the singular value difference spectrum to accurately determine the noise reduction order [18,19], it can effectively eliminate the interference of noise components in the signal and retain the main components of fault information. Meng et al. [20] proposed a fault diagnosis method based on improved variational modal decomposition (VMD) and spectrum reconstruction, which effectively suppresses noise interference. Wang et al. [21] proposed a fault diagnosis method based on empirical wavelet transform and singular value decomposition for high-speed train rolling bearings, applied to fault diagnosis, but it depends on the selection of the EWT parameters. Ma et al. [22] applied the VMD method optimized by RIME for rolling bearing fault diagnosis by incorporating a variational constraint. This method transforms the process of obtaining signal components into a variational solution problem, using the alternating direction multiplier method to continuously update each mode and its central frequency, gradually demodulating each mode to the corresponding baseband, and achieving noise reduction decomposition of the original signal [23,24,25,26]. VMD has the following advantages [27,28,29,30]: (1) VMD, through multiple adaptive Wiener filtering, exhibits better noise robustness. (2) By properly controlling the convergence conditions, VMD has a smaller sampling effect. (3) In mode separation, VMD can successfully separate two signals with close frequencies. (4) The endpoint effect of VMD is much weaker than that of recursive mode decomposition, and its computational efficiency is high. Dung Beetle Optimization (DBO) is a novel swarm intelligence optimization algorithm proposed by Jiankai Xue and Bo Shen [31] in 2022. The DBO algorithm simulates the foraging behavior of dung beetles, including behaviors such as rolling balls, dancing, foraging, stealing, and breeding, which are translated into search strategies in the algorithm to solve optimization problems. Compared with PSO, GWO, and other meta-heuristics, DBO offers faster convergence, stronger global search capability, and fewer hyper-parameters, making it particularly suitable for the non-convex, multi-modal optimization of VMD parameters. This study applies it to optimize VMD parameters due to its excellent global search ability and faster convergence speed. By using the intelligent DBO algorithm to address issues such as VMD parameter selection, mode mixing, convergence speed, and global search, the performance and application range of VMD are improved.

Recently, several studies have explored the combination of DBO, VMD, and SVD for signal denoising in other domains. Chen et al. [32] proposed a DBO–VMD–SVD method for underwater acoustic signal denoising, while Song et al. [33] developed a VMD-SVD-WTD method based on an improved DBO for transient electromagnetic noise reduction. While these works share similar technical components, there are three fundamental differences. First, the sequence of integration differs: previous methods apply DBO-VMD first followed by SVD, whereas the present method employs SVD as a preprocessing step before DBO-VMD, which is specifically designed to enhance periodic impulse structures. Second, the IMF selection criteria are distinct: the proposed method uses a joint kurtosis-peak-to-peak criterion specifically designed for the impulsive nature of bearing fault signals. Third, the application domain and objective are different: previous combinations were developed for underwater acoustic and electromagnetic noise reduction, while the proposed framework is tailored for rolling bearing fault feature extraction, with the explicit goal of preserving and enhancing fault-related periodic impulses for Hilbert envelope spectrum analysis.

Unlike a simple sequential integration of existing techniques, the proposed framework establishes a synergistic mechanism specifically designed for rolling bearing fault characteristics: (1) the adaptive SVD via singular value difference spectrum not only suppresses broadband noise but also enhances the periodic impulse structure, providing a preconditioned input that improves VMD’s convergence and mode separation; (2) the DBO algorithm employs minimum envelope entropy as the fitness function, which is physically tailored to impulsive fault signals rather than generic optimization objectives, enabling non-convex parameter search in VMD; and (3) the kurtosis-peak-to-peak joint criterion mitigates the energy-decay bias inherent in single-index screening, ensuring robust IMF selection across varying fault severities. These three modules are mutually reinforcing rather than independent preprocessing steps.

The remaining parts of the paper are organized as follows: Section 2 introduces the basic theories of VMD, SVD, and DBO. Section 3 proposes the design of the fault feature extraction method based on SVD-DBO-VMD. Section 4 is the experiment and result analysis, which verifies the extraction effect of the proposed method. Section 5 presents the findings and conclusions of this research.

## 2. Materials and Methods

### 2.1. Variational Mode Decomposition (VMD)

VMD is a multi-component signal adaptive decomposition method that can decompose complex signals into AM-FM IMFs.

Selecting a suitable parameter can effectively suppress modal aliasing. VMD is fundamentally different from the layer-by-layer screening mode adopted by traditional EMD and has solid theoretical support. Its overall structure involves constructing and solving variational problems, which mainly involve three important concepts: classical Wiener filtering, Hilbert transform, and frequency mixing. VMD uses Wiener filtering for denoising and obtains the center frequency of AM-FM IMFs by initialization through setting the limited bandwidth parameter and the center frequency. Then, the modal function of each IMF is obtained according to different center frequencies.

#### 2.1.1. The Construction of Variational Problems Solutions to Variational Problems

The variational problem aims to decompose the input signal $x\left(t\right)$ into K IMF components $u\left(t\right)$, and then obtain its analytical signal through Hilbert transform for each component $u\left(t\right)$. Mixing the analytical signal with the estimated center frequency $w_{k}$, under the constraint that the sum of each component is equal to the signal $x\left(t\right)$, the variational problem is as follows:

(1) $$\left\{\begin{array}{l} \underset{\left\{u_{k}\right\},\left\{w_{k}\right\}}{\min}\left\{\sum_{k}\|\partial_{t}\left[\left(\delta \left(t\right)+\frac{j}{\pi t}\right)u_{k}\left(t\right)\right]e^{-jw_{k}t}{\|}_{2}^{2}\right\}\\ s.t\;\sum_{k}u_{k}=x\left(t\right) \end{array}\right.$$

where $\partial_{t}$ represents the partial derivative with respect to t, and $\delta \left(t\right)$ represents the Dirac delta function.

#### 2.1.2. Solution of Variational Problems

Using the Lagrangian multiplication operator $\lambda_{t}$ and the quadratic penalty factor α, the constrained variational problem is transformed into an unconstrained variational problem as follows:

(2) $$L\left(\left\{u_{k}\right\},\left\{w_{k}\right\},\lambda \right)=\alpha \sum_{k}\|\partial_{t}\left[\left(\delta \left(t\right)+\frac{j}{\pi t}\right)u_{k}\left(t\right)\right]e^{-ju_{k}t}{\|}_{2}^{2}\;+\|x\left(t\right)-\sum_{k}u_{k}\left(t\right){\|}_{2}^{2}+\langle \lambda \left(t\right),x\left(t\right)-\sum_{k}u_{k}\left(t\right)\rangle$$

The alternate direction method of multipliers (ADMM) is used to find the saddle point of the Lagrangian function for the optimal solution of Equation (1). The specific implementation steps are as follows [22]:

Step 1: Initialize $\left\{{\hat{u}}_{k}^{1}\right\}$, $\left\{{\hat{w}}_{k}^{1}\right\}$, ${\hat{\lambda }}^{1}$, n.

Step 2: Execute the loop n=n+1.

Step 3: For all w>0, update ${\hat{u}}_{k}$.

(3) $${\hat{u}}_{k}^{n+1}\left(w\right)\leftarrow \frac{\hat{x}\left(w\right)-\sum_{i<k}{\hat{u}}_{i}^{n+1}\left(w\right)-\sum_{i<k}{\hat{u}}_{i}^{n}\left(w\right)+\frac{{\hat{\lambda }}^{n}\left(w\right)}{2}}{1+2\alpha {\left(w-w_{k}^{n}\right)}^{2}},\;k\in \left\{1,K\right\}$$

Step 4: Update $w_{k}$.

(4) $$w_{k}^{n+1}\leftarrow \frac{\int_{0}^{\infty }w{\left|{\hat{u}}_{k}^{n+1}\left(w\right)\right|}^{2}dw}{\int_{0}^{\infty }{\left|{\hat{u}}_{k}^{n+1}\left(w\right)\right|}^{2}dw}\;k\in \left\{1,K\right\}$$

Step 5: Update λ.

(5) $${\hat{\lambda }}^{n+1}\left(w\right)\leftarrow {\hat{\lambda }}^{n}\left(w\right)+\tau \left(\hat{x}\left(w\right)-\sum_{k}{\hat{u}}_{k}^{n+1}\left(w\right)\right)$$

Step 6: Repeat steps (2) to (5) until Equation (6) is satisfied to stop the iteration and obtain K IMF components.

(6) $$\sum_{k}\|{\hat{u}}_{k}^{n+1}-{\hat{u}}_{k}^{n}{\|}_{2}^{2}/\|{\hat{u}}_{k}^{n}{\|}_{2}^{2}<\varepsilon$$

The VMD algorithm is summarized as follows [27]: (1) each mode is directly updated continuously in the frequency domain and finally converted to the time domain by Fourier inversion; (2) as the center of gravity of the power spectrum of each mode, the center frequency is re-predicted and updated cyclically in this way.

### 2.2. Singular Value Decomposition (SVD)

Assuming that the measured group of rolling bearings contains noise signal $x_{1},x_{2},x_{3},\cdots ,x_{q}$, based on the phase space reconstruction theory [31,33], the Hankel matrix of p×q dimension can be obtained as follows:

(7) $$H_{p}=\left[\begin{array}{cccc} x_{1} & x_{2} & \cdots & x_{q}\\ x_{2} & x_{3} & \cdots & x_{q+1}\\ \vdots & \vdots & \vdots & \vdots \\ x_{p} & x_{p+1} & \cdots & x_{p+q-1} \end{array}\right]$$

where $H_{p}$ is a matrix of order p×q, p+q−1 is the signal length and p>q.

Singular value decomposition of $H_{p}$ can be obtained as follows:

(8) $$H_{p}=U\Lambda V^{T}$$

where U and V are p×p and q×q orthogonal matrices, Λ is the diagonal array of p×q with diagonal elements $\sigma_{1},\sigma_{2},\ldots ,\sigma_{m}$, $m=\min\left(p,q\right)$, $\sigma_{1}\geq \sigma_{2}\geq \cdots \geq \sigma_{m}$, and $\sigma_{1},\sigma_{2},\ldots ,\sigma_{m}$ are called singular values of $H_{p}$.

Assuming that $r\left(H_{p}\right)=k\left(k<p\right)$ of $H_{p}$, the conclusion can be drawn according to the singularity theory and the best approximation theorem for matrices in the sense of Frobeious parametrization. If the first k singular values of $H_{p}$ are retained while the noise corresponds to the smaller singular values set to 0 later, and then the inverse process of singular value decomposition is used to obtain a matrix, denoted as $H_{p}^{\prime }$, then $H_{p}^{\prime }$ is the best approximation matrix of $H_{p}$($r\left(H_{p}\right)=k\left(k<p\right)$) [34], which reduces the noise and improves the signal-to-noise ratio.

The key issue of SVD noise reduction is to choose a reasonable noise reduction order. The noise reduction order has a significant impact on the signal noise reduction effect. In order to select a reasonable noise reduction order and describe the sudden change of singular values, this paper introduces the concept of singular value difference spectrum [35], which is defined as follows:

(9) $$\varepsilon_{i}=\sigma_{i}-\sigma_{i+1},\;i=1,2,\ldots ,p-1$$

For quantitative evaluation of the denoising performance, the signal-to-noise ratio (SNR) is calculated based on the energy partition between the dominant structured components and the residual random noise. Specifically, the SNR is computed via spectral estimation as the ratio of the power of dominant frequency components (corresponding to the periodic impulse structures preserved by SVD) to the power of the residual broadband background noise. This approach does not require an explicit clean reference signal and is consistent with the physical mechanism of SVD denoising, where large singular values retain the signal subspace and small singular values correspond to the noise subspace.

### 2.3. Dung Beetle Optimization (DBO)

DBO is a novel optimization algorithm based on the behavior of dung beetles [31], characterized by rapid convergence, high precision, and high stability. The DBO algorithm primarily obtains the global optimal position and fitness value through the dung beetles’ positional changes, featuring five behaviors: dancing, rolling, stealing, foraging, and breeding. Let n represent the population size of the dung beetles.

The position update and definition when the dung beetle rolls the ball forward:

(10) $$y_{i}^{t+1}=y_{i}^{t}+gGy_{i}^{t-1}+h\left|y_{i}^{t}-y_{worst}^{t}\right|$$

where $y_{i}^{t}$ is the location of the i dung beetle after t iterations; g is a random number −1 or 1; G is a random number within (0, 0.2); $y_{worst}^{t}$ is the global worst position; and h is a random number within (0, 1).

When the dung beetle encounters an obstacle and cannot proceed, it needs to readjust itself through dancing to obtain a new route.

(11) $$y_{i}^{t+1}=y_{i}^{t}+\tan\theta \left|y_{i}^{t}-y_{i}^{t-1}\right|$$

where θ is a random number in [0, π], and when θ=0,π/2,π, there is no change in the dung beetle position.

Formula for the reproduction process of dung beetles:

(12) $$y_{i}^{t+1}=y_{gbest}^{t}+b_{1}\left(y_{i}^{t}-L_{b}^{*}\right)+b_{2}\left|y_{i}^{t}-U_{b}^{*}\right|$$

where $b_{1}$ and $b_{2}$ are both an independent 1×b dimensional random matrix; $L_{b}^{*}$ and $U_{b}^{*}$ are the upper and lower bounds of the propagation region, respectively; and $y_{gbest}^{t}$ is the current global best position.

Update the existing position of a dung beetle while foraging and define it as:

(13) $$y_{i}^{t+1}=y_{i}^{t}+C_{1}\left(y_{i}^{t}-L_{b}^{l}\right)+C_{2}\left|y_{i}^{t}+U_{b}^{l}\right|$$

where $L_{b}^{l}$ and $U_{b}^{l}$ are the upper and lower boundaries of the foraging area, respectively; $C_{1}$ is a 1×b dimensional matrix with normal distribution; and $C_{2}$ is a 1×b dimensional random matrix.

In dung beetle populations, there are some dung beetles that steal dung balls from other dung beetles, and the updated equation for the location of the stealing dung beetles is

(14) $$y_{i}^{t+1}=y_{lbest}^{t}+Sg\left(\left|y_{i}^{t}-y_{gbest}^{t}\right|+\left|y_{i}^{t}-y_{lbest}^{t}\right|\right)$$

where g is a normally distributed 1×b dimensional matrix; $y_{lbest}^{t}$ is the local optimum position; and S is a constant.

## 3. Weak Fault Feature Extraction Method Based on SVD-DBO-VMD

### 3.1. Evaluation Indicators

Kurtosis is a dimensionless parameter that is particularly sensitive to impact pulses and pulse-like fault signals. Therefore, it is especially suitable for the diagnosis of surface damage faults, especially early faults. Peak-to-peak value, as another key evaluation index of bearing vibration signals, provides an intuitive reflection of the bearing operating state. It quantifies the overall amplitude of bearing vibration by measuring the difference between the maximum peak and the minimum peak in the vibration signal. When rolling element bearings fail, they are usually accompanied by an increase in impact pulses, leading to changes in signal amplitude. These two indicators are particularly sensitive to the impact pulses of bearing faults. Utilizing their characteristics to filter the data can not only reduce the computational complexity of feature extraction but also achieve the purpose of data denoising.

### 3.2. Design of Feature Extraction Method Based on SVD-DBO-VMD

**Figure 1:**
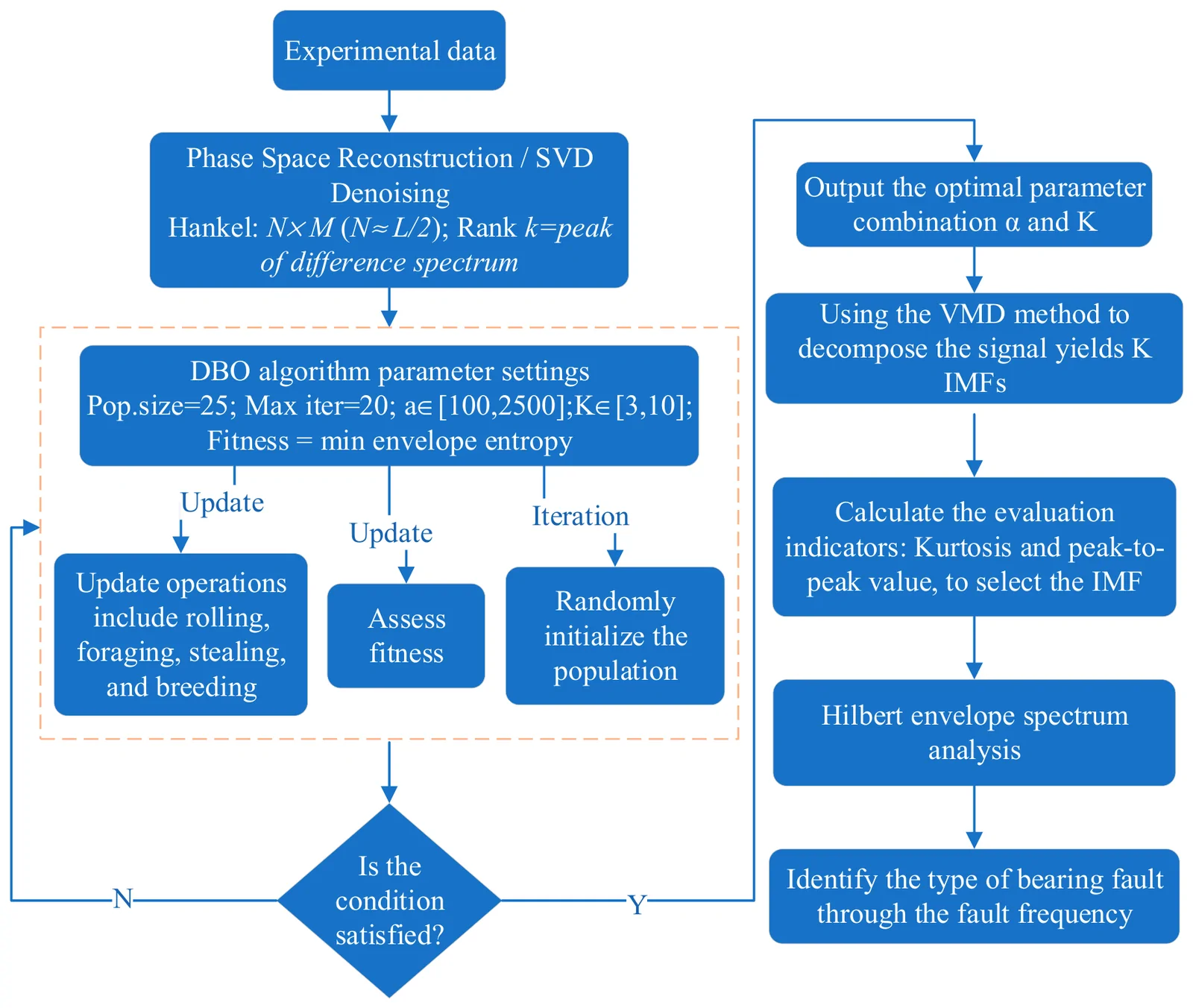
Flowchart of SVD-DBO-VMD fault feature extraction.

The proposed method utilizes SVD-DBO-VMD to effectively extract sensitive features and improve the signal-to-noise ratio, highlighting the impact pulses in the original signal. This allows the IMF components selected after signal denoising to have a clearer physical meaning, which is conducive to accurately analyzing the fault frequency of rolling bearing working conditions through the envelope spectrum, as shown in Figure 1. The detailed steps are as follows:

Step 1 Phase Space Reconstruction: Perform phase space reconstruction on the original vibration signal. Based on the singular value difference spectrum graph, find the abrupt change point of the difference value and use it as the optimal order for SVD to reduce the impact of noise and improve the quality of signal decomposition.

Step 2 Parameter Optimization with DBO: Input the denoised signal, set the range of VMD parameters (penalty factors α and K upper and lower limits), define the DBO algorithm’s fitness function, dimensions, maximum number of iterations, and dung beetle population size. Simulate the dung beetle rolling dung balls to update parameters and guide the population towards the optimal solution based on the minimum value of the fitness function (minimum envelope entropy). Select individuals with high adaptability for reproduction, update the positions of individuals in the population, and stop when the maximum number of iterations is satisfied. Output the optimal parameter combination α and K.

Step 3 VMD: Set the optimal parameter combination for the VMD algorithm, decompose the denoised signal, and obtain K IMF components. Based on the component evaluation indicators, calculate the kurtosis and peak-to-peak value of each IMF component, and select an IMF component that contains the most fault information as a sensitive feature.

Step 4 Hilbert Envelope Spectrum Analysis: Perform Hilbert transformation on the sensitive feature signal, take the extremum to form one-dimensional data, and then perform FFT transformation to obtain the signal envelope spectrum graph. Judge the type of fault in the rolling bearing through the fault frequency in the Hilbert envelope spectrum graph.

## 4. Experiment and Result Analysis

To verify the effectiveness of the method in Section 3, the research dataset used in this study is the fault data from the Bearing Data Center of Case Western Reserve University (CWRU) in the United States [36] and the bearing fault dataset from Paderborn University (PU) in Germany [37].

### 4.1. CWRU Data Experiment Analysis

The bearing tested on the CWRU experimental rig is the SKF 6205 deep groove ball bearing at the drive end, with specific parameters shown in [36]. The working mode of the bearing is that the inner ring rotates with the shaft, while the outer ring is fixed on the housing. The bearing rotation speed is 1796 rpm, the sampling frequency is 12 kHz, and the sampling length is taken as 2048. The local pitting damage size of the bearing’s inner ring, outer ring, and rolling elements is 0.007×0.011 inches, which is artificially made by an electric discharge machine. The measurements are taken by vibration acceleration sensors installed on the induction motor (Table 1).

The types of bearing failures include rolling element faults, inner ring faults, and outer ring faults. This study takes the outer ring fault signal caused by pitting damage as an example to provide a detailed explanation of the fault-sensitive feature extraction method for rolling bearings. According to the bearing fault frequency formula, the outer ring frequency is calculated to be 104.57 Hz.

**Figure 2:**
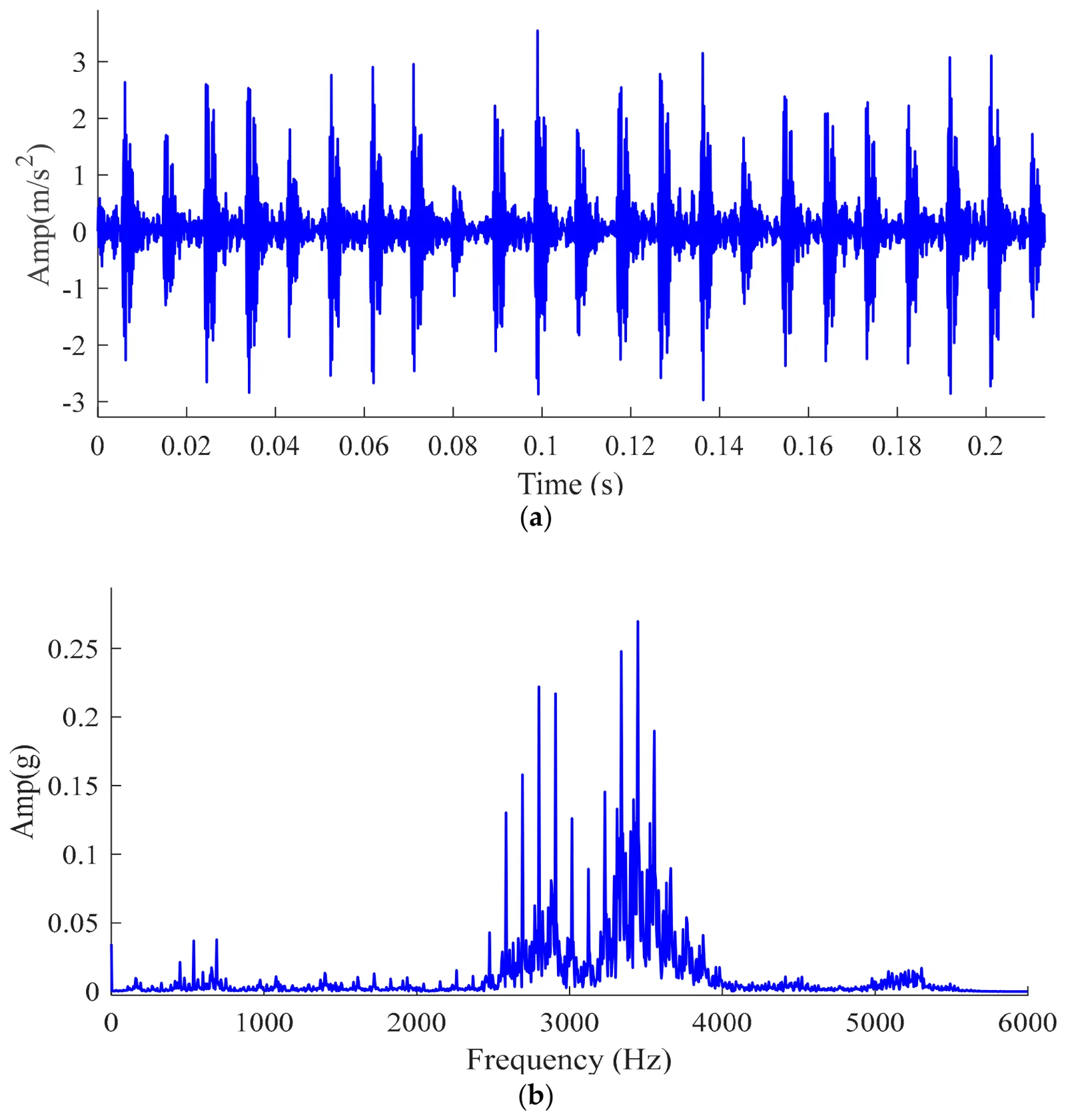
Outer ring waveform diagram: (**a**) Outer ring time domain waveform diagram; (**b**) outer ring frequency domain waveform diagram.

The time-frequency domain waveform of the outer ring fault is shown in Figure 2. From the spectrum in Figure 2b, it can be seen that the characteristic frequency of the outer ring fault has undergone severe migration, the 0–2000 Hz band contains shaft rotational frequency and its harmonics; the 2500–4000 Hz band exhibits elevated energy likely related to bearing resonances modulated by the outer race defect frequency (104.57 Hz) and its harmonics; the 4500–5500 Hz band corresponds to structural resonance. Although periodic impacts are visible in the time domain, the broadband noise and complex spectral components degrade the subsequent VMD quality. SVD is employed to improve the overall SNR and decomposition fidelity, rather than merely making the fault frequency visible.

**Figure 3:**
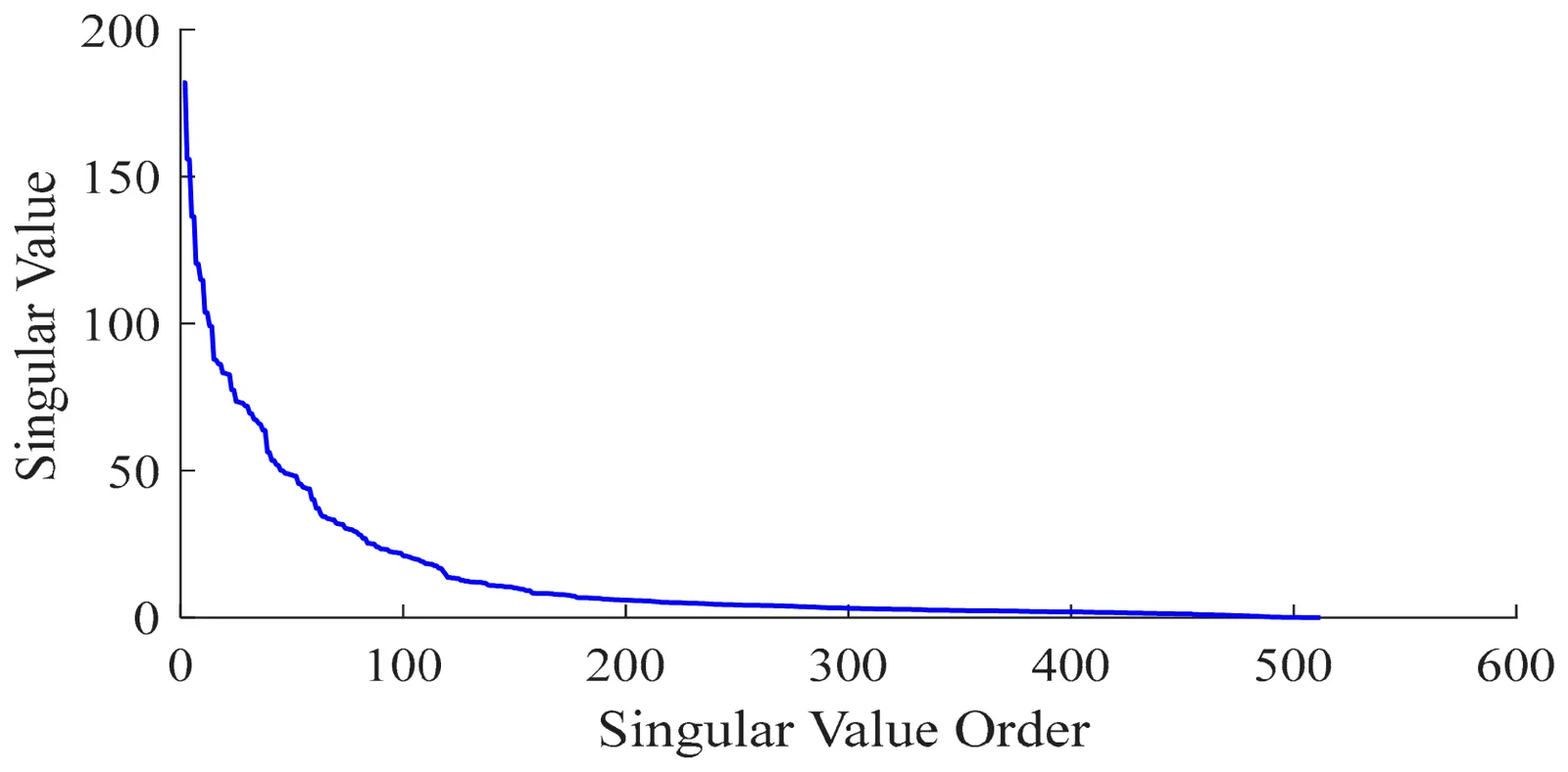
Distribution of singular values of faults in the outer ring.

**Figure 4:**
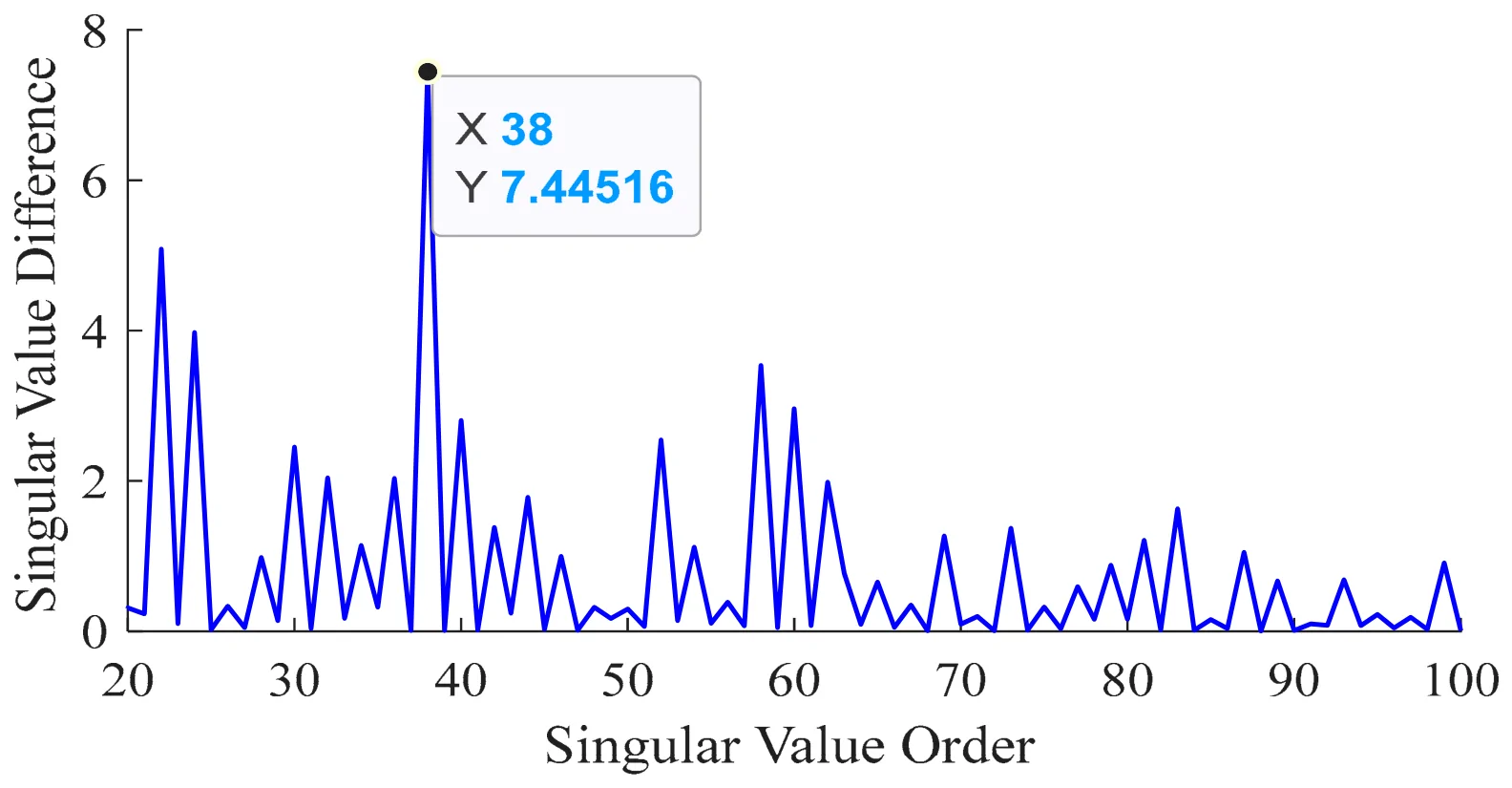
Outer ring fault singular value difference spectrum.

**Figure 5:**
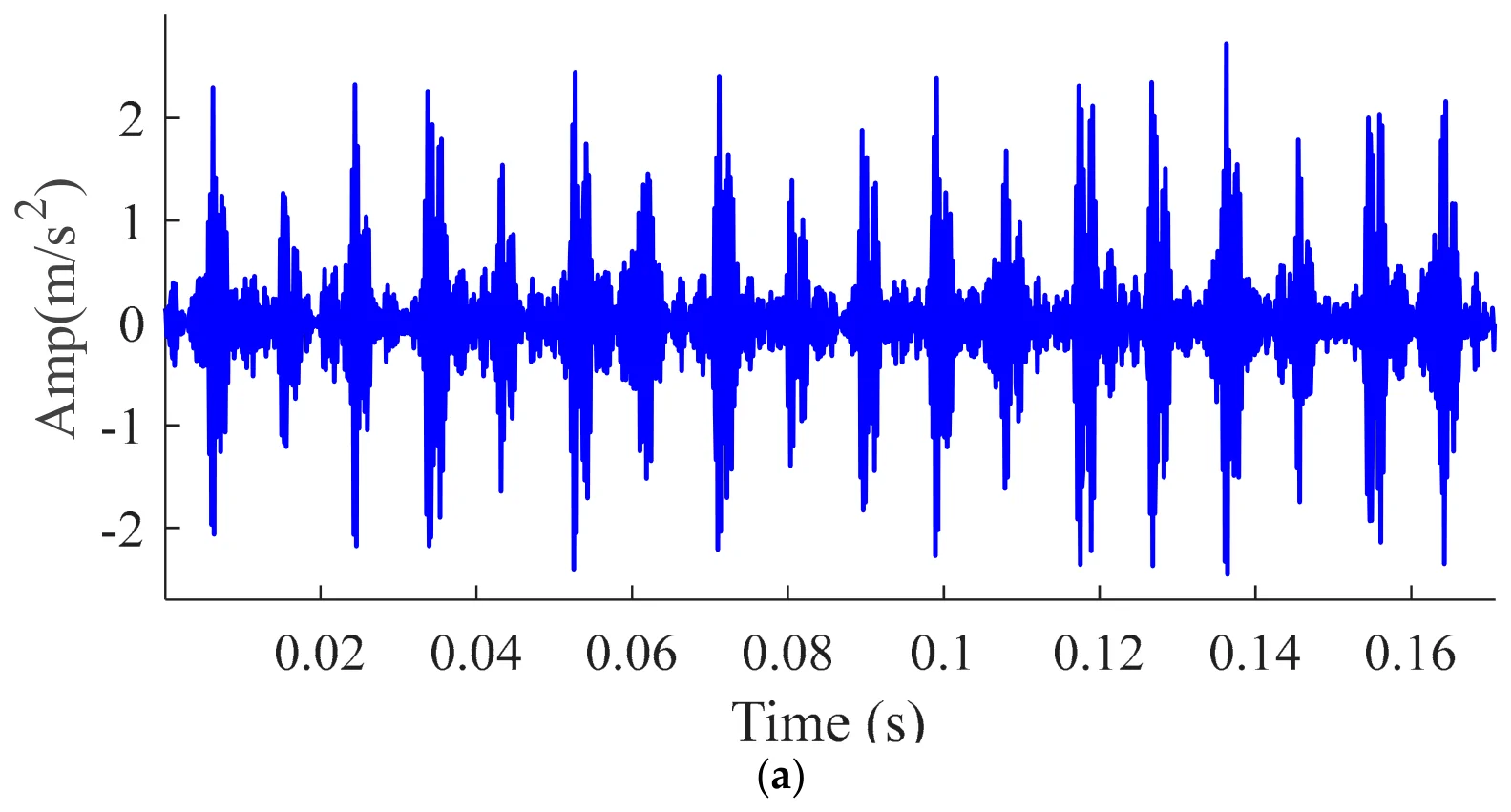

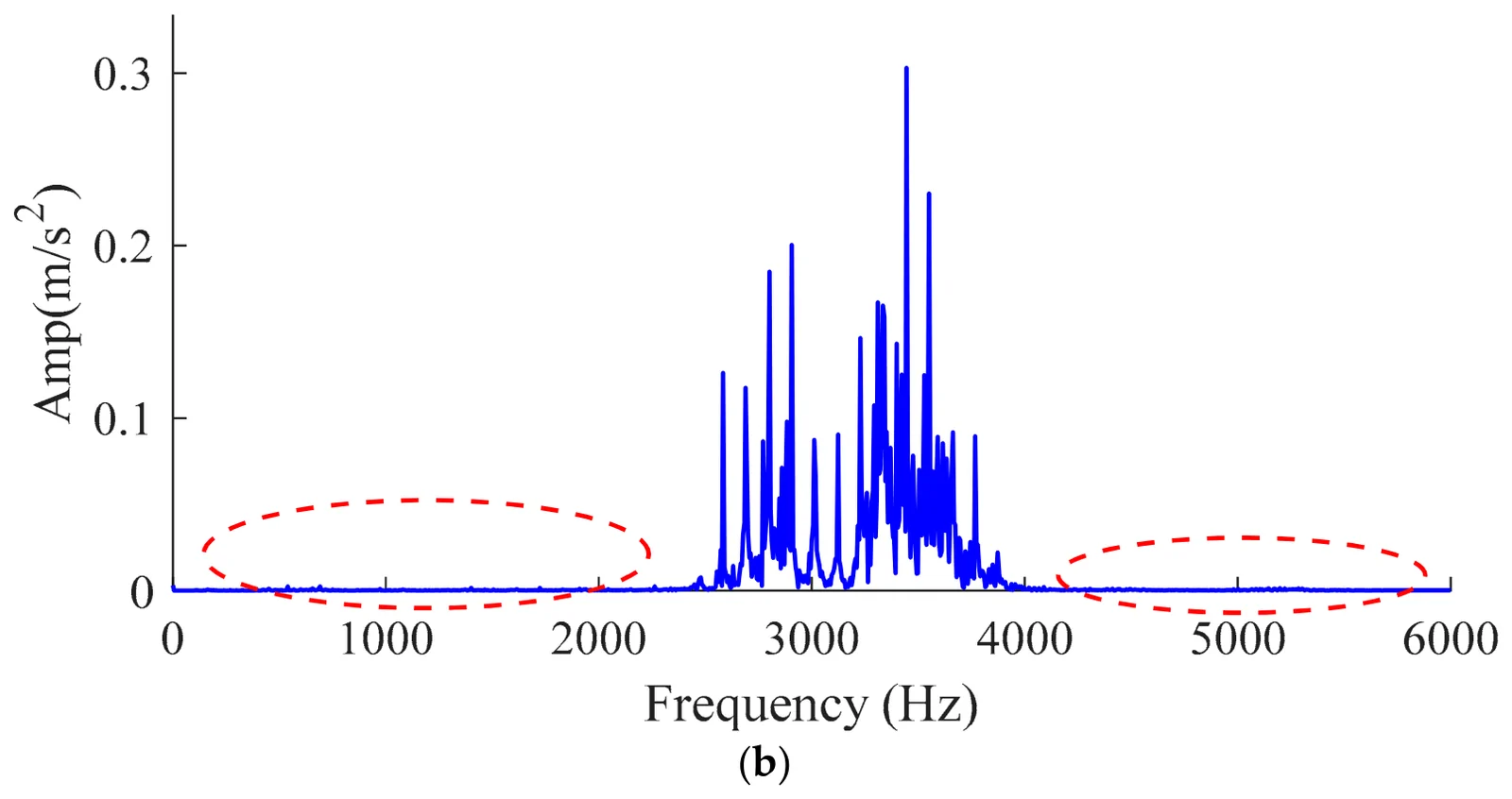
Time domain and frequency domain waveforms after SVD denoising: (**a**) Time waveform after SVD denoising; (**b**) frequency waveform after SVD denoising.

Using SVD for denoising preprocessing of the outer ring fault signal, the distribution of singular values and the singular value difference spectrum are shown in Figure 3 and Figure 4. Due to the rapid decay of singular values, Figure 4 presents a partial view of the first 100 orders to clearly identify the abrupt change point. Selecting the appropriate denoising order is crucial, as different denoising orders result in different signal denoising effects. When the singular value sequence number is small, the difference value is large, indicating that this singular value represents the main component of the signal; when the difference value decreases, it indicates that noise is dominant. From Figure 4, it can be concluded that the peak value in the difference spectrum is 38, which is used as the SVD denoising order. The denoising result is shown in Figure 5.

**Figure 6:**
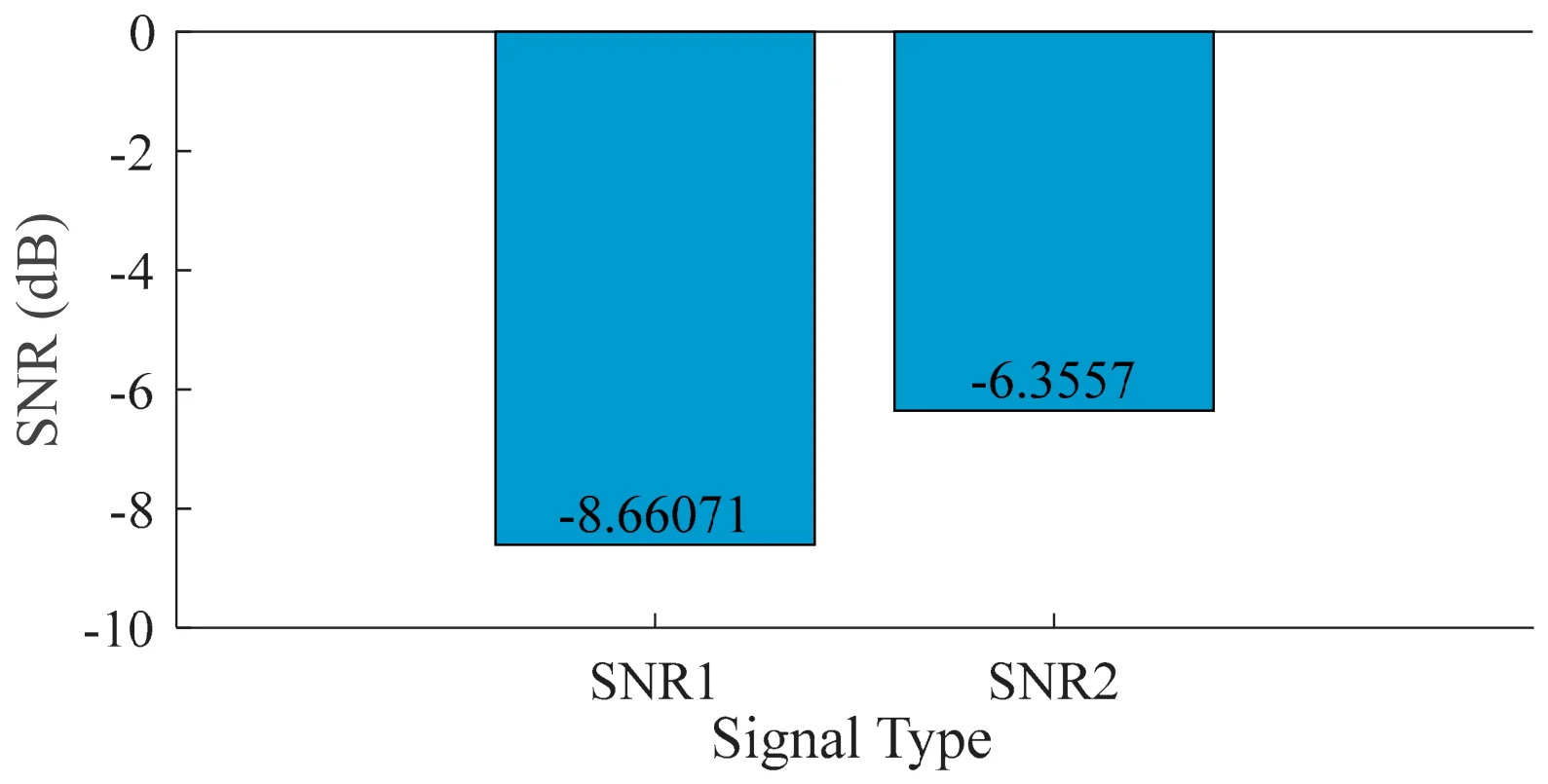
Comparison chart of SNR before and after noise reduction of the outer ring.

Comparing the denoised signal (Figure 5) with the undenoised signal (Figure 2), it can be observed that unwanted frequency components and background interference have been effectively attenuated and the dominant signal energy is concentrated in the lower frequency band. However, some high-frequency bearing resonance information may also be attenuated. The SVD denoising primarily retains the principal components associated with the periodic impulse train, which is sufficient for subsequent VMD and envelope analysis. To further verify the denoising effect, the signal-to-noise ratio (SNR) of the original signal (Figure 2, denoted as SNR1) and the SVD-denoised signal (Figure 5, denoted as SNR2) were calculated respectively via spectral estimation following the procedure described in Section 2.2, as shown in Figure 6.

From Figure 6, it can be seen that SNR1 = −8.6071 dB, and SNR2 = −6.3557 dB. SNR2 has a higher signal-to-noise ratio than SNR1, indicating that the signal quality is significantly better than that of SNR1. The signal has lower noise interference and higher clarity, achieving the expected noise reduction effect.

Due to the multi-component modulated nature of the signal, it is necessary to optimize the parameters for VMD to find the best ones. In this paper, the DBO (Dung Beetle Optimization) algorithm is used to optimize the parameter settings as shown in Table 2. By simulating the behavior of dung beetles, with the minimum envelope entropy as the fitness function, the optimal mode number K and penalty factor A of VMD are optimized. Utilizing its global search capability, the best parameter combination for decomposition is found, which improves the quality and accuracy of the signal.

**Figure 7:**
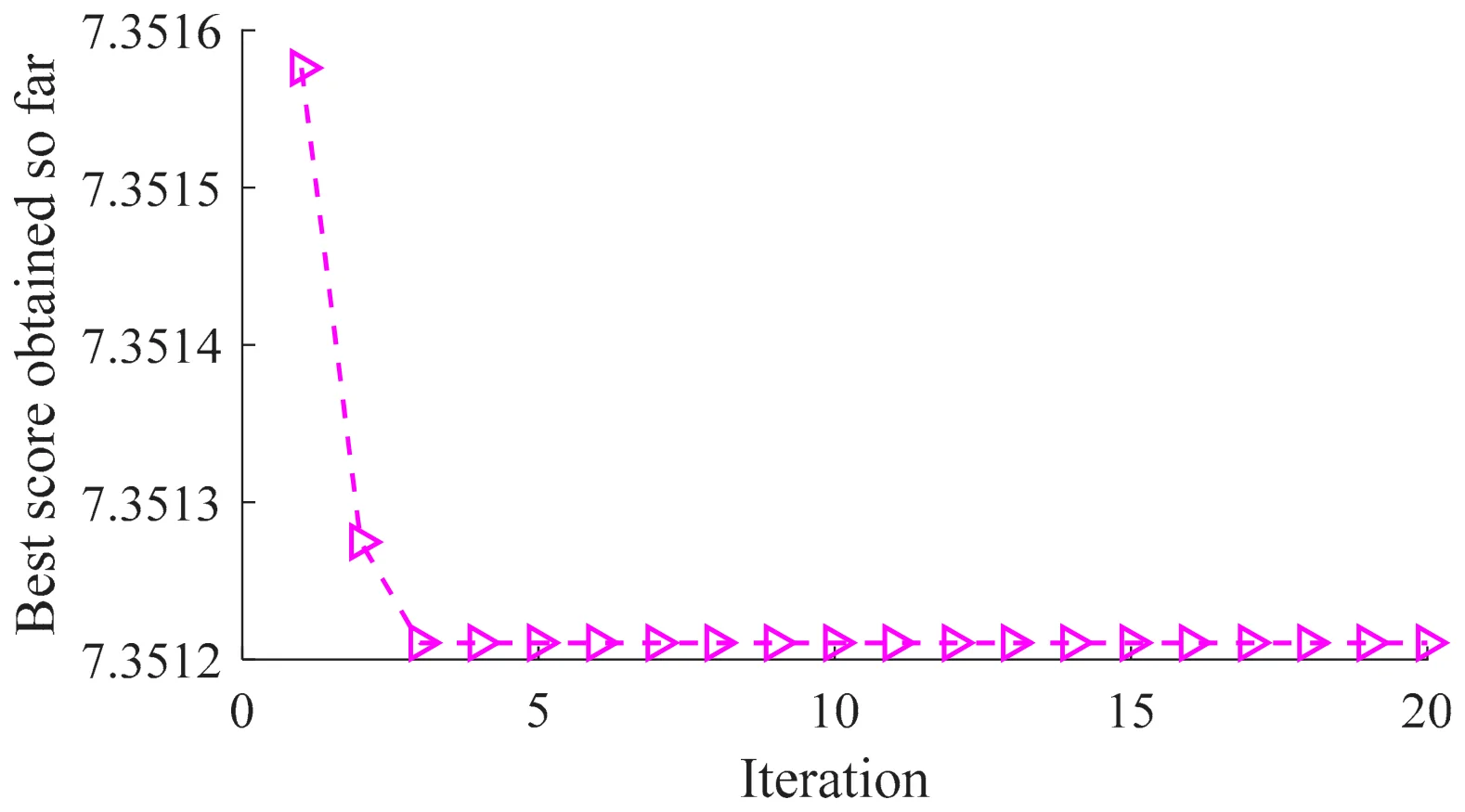
Iterative plot of VMD parameters for DBO of outer ring data.

**Figure 8:**
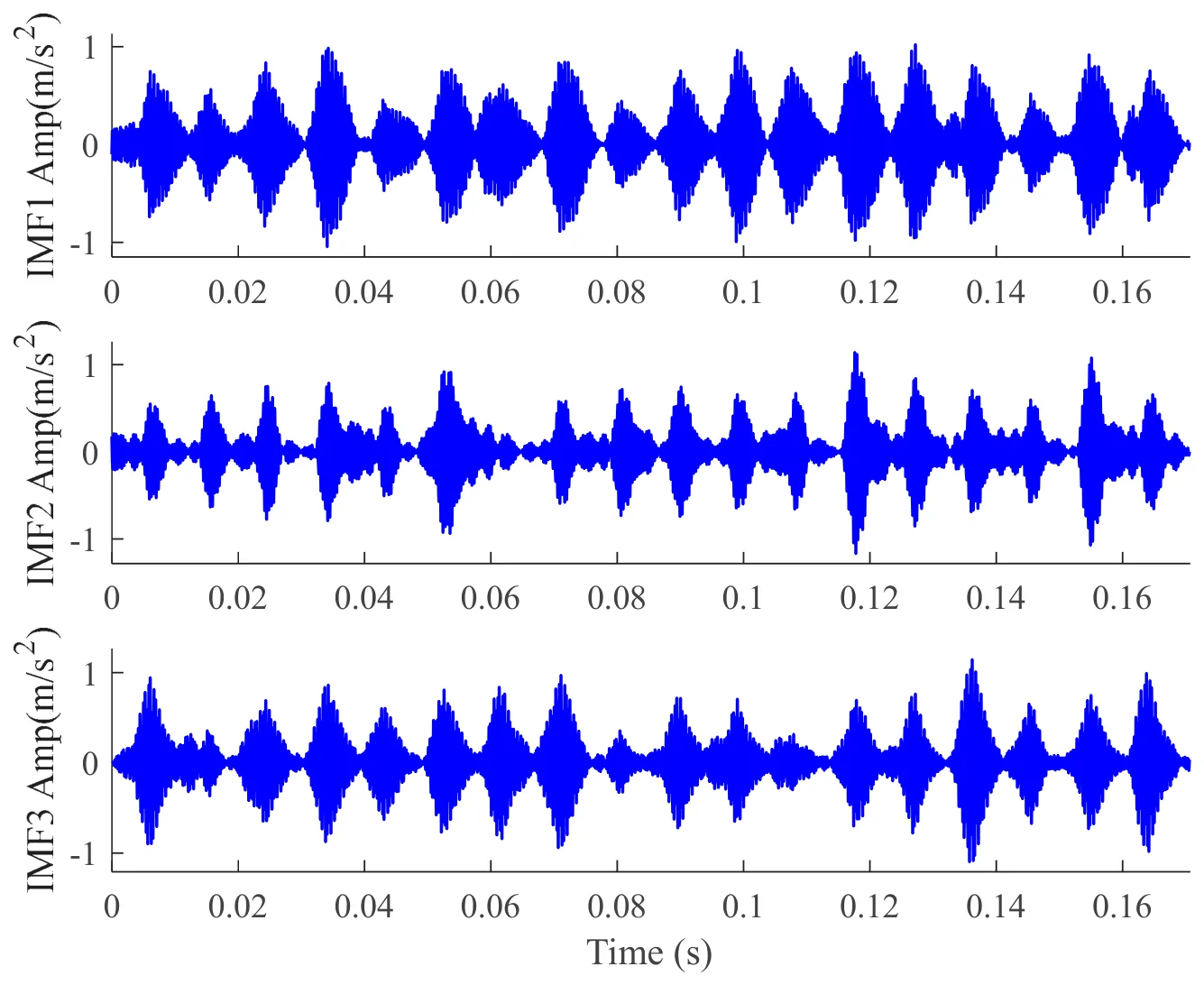
VMD of outer ring fault signal after SVD noise reduction.

From Figure 7, it can be seen that the fitness function converges rapidly within the first few iterations and stabilizes after approximately 3 iterations, with the optimal fitness function value being 7.3512, and the best parameter combination for VMD is K=3 and α=277. The pitting fault signal of the outer ring, after being decomposed by VMD with the set parameters, results in 3 IMF components as shown in Figure 8.

**Figure 9:**
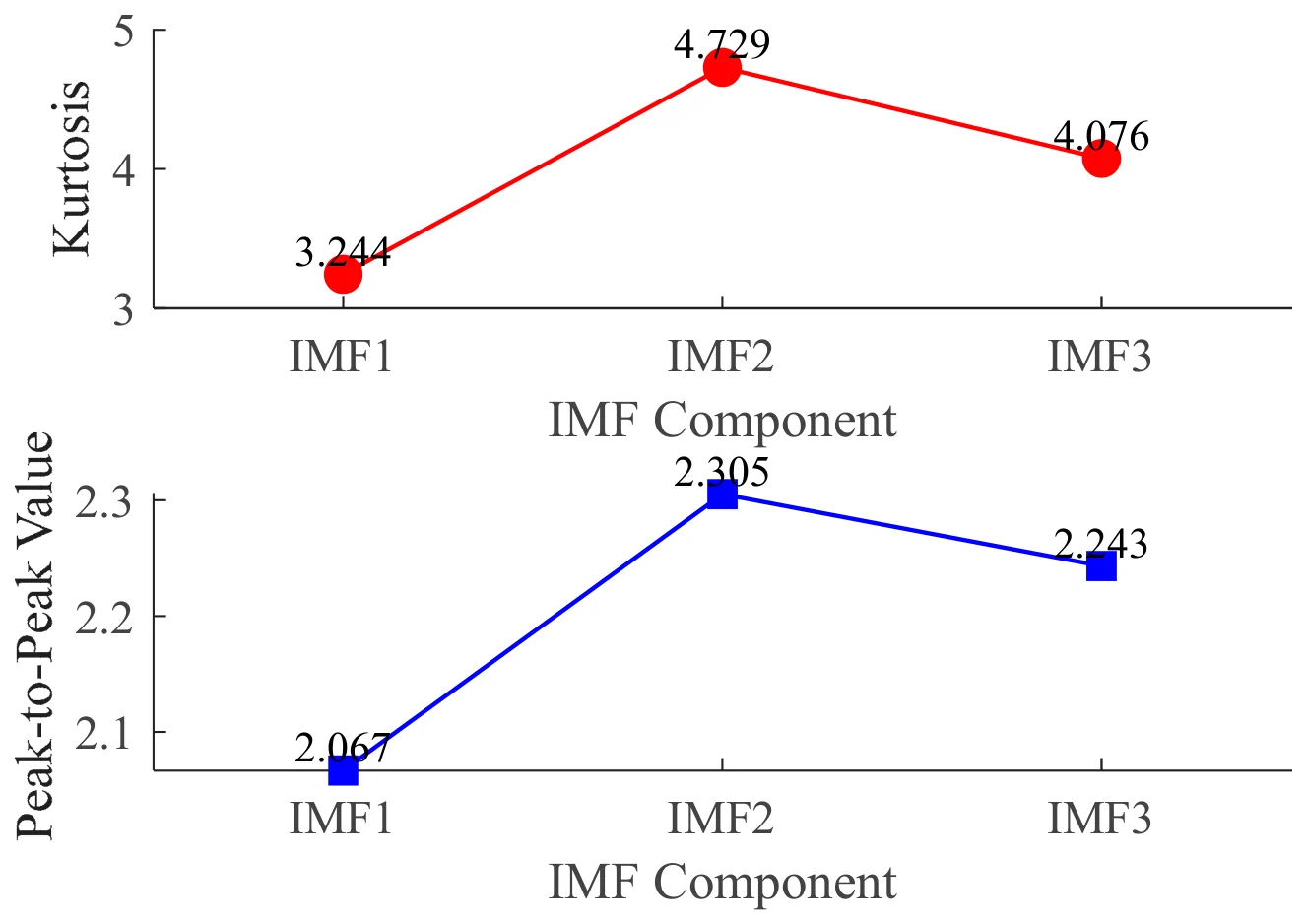
Graph of two evaluation indicators for each IMF component in the outer ring.

From the 3 components obtained by VMD, select an IMF that contains the most fault information as a sensitive feature. In this paper, evaluation indicators are used for screening, that is, by calculating the kurtosis and peak-to-peak value of each IMF component, an IMF component with the richest fault information is selected. Figure 9 shows the two evaluation indicators for each IMF component of the outer ring fault.

The fault frequency of vibration signals is often contained within the high-frequency IMF components, while the remaining IMF components are mostly considered as noise signals. From Figure 9, IMF2 has the highest kurtosis and peak-to-peak values and was therefore selected as the sensitive component for the Hilbert envelope spectrum analysis of bearing outer ring fault frequencies.

**Figure 10:**
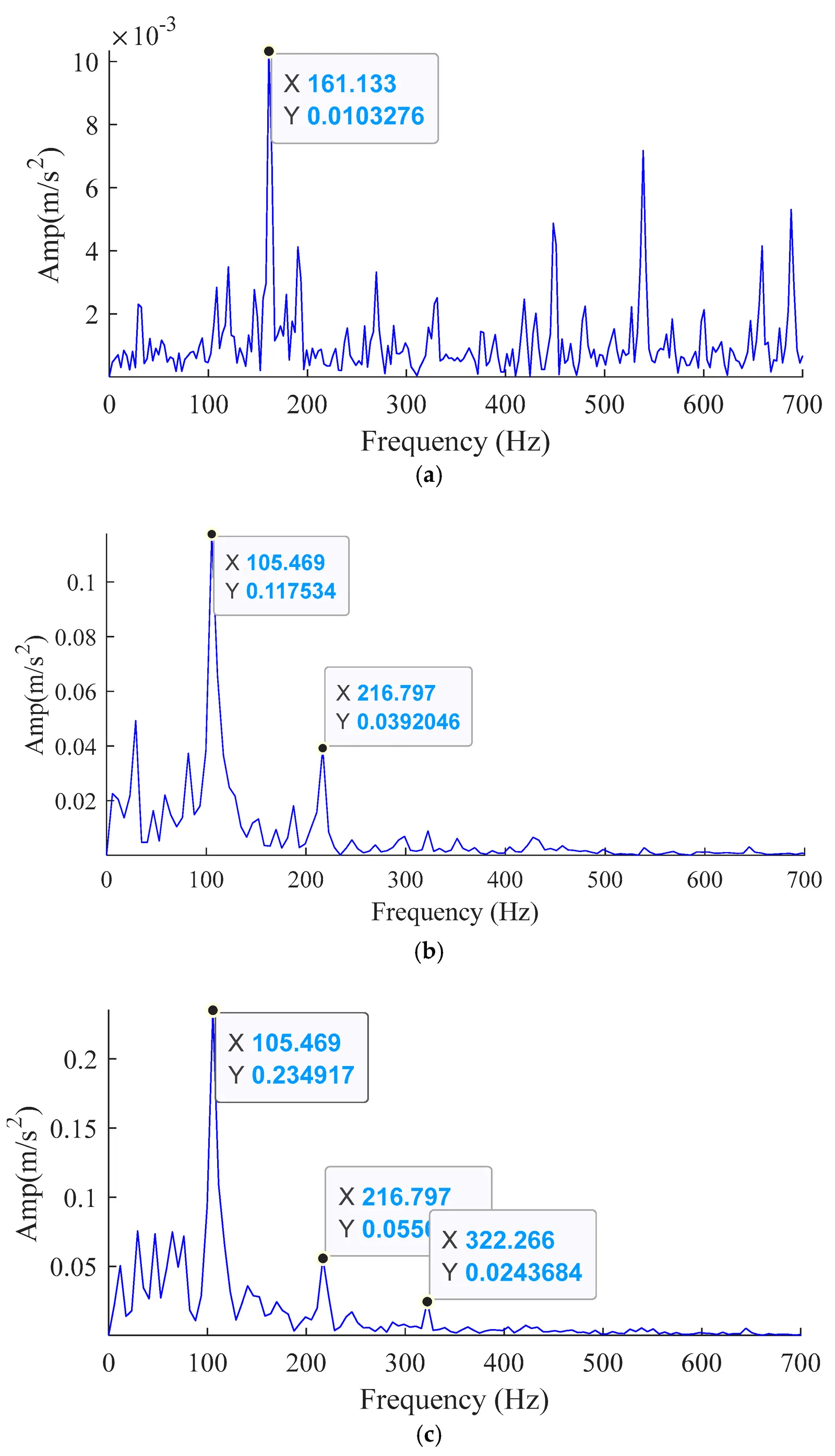

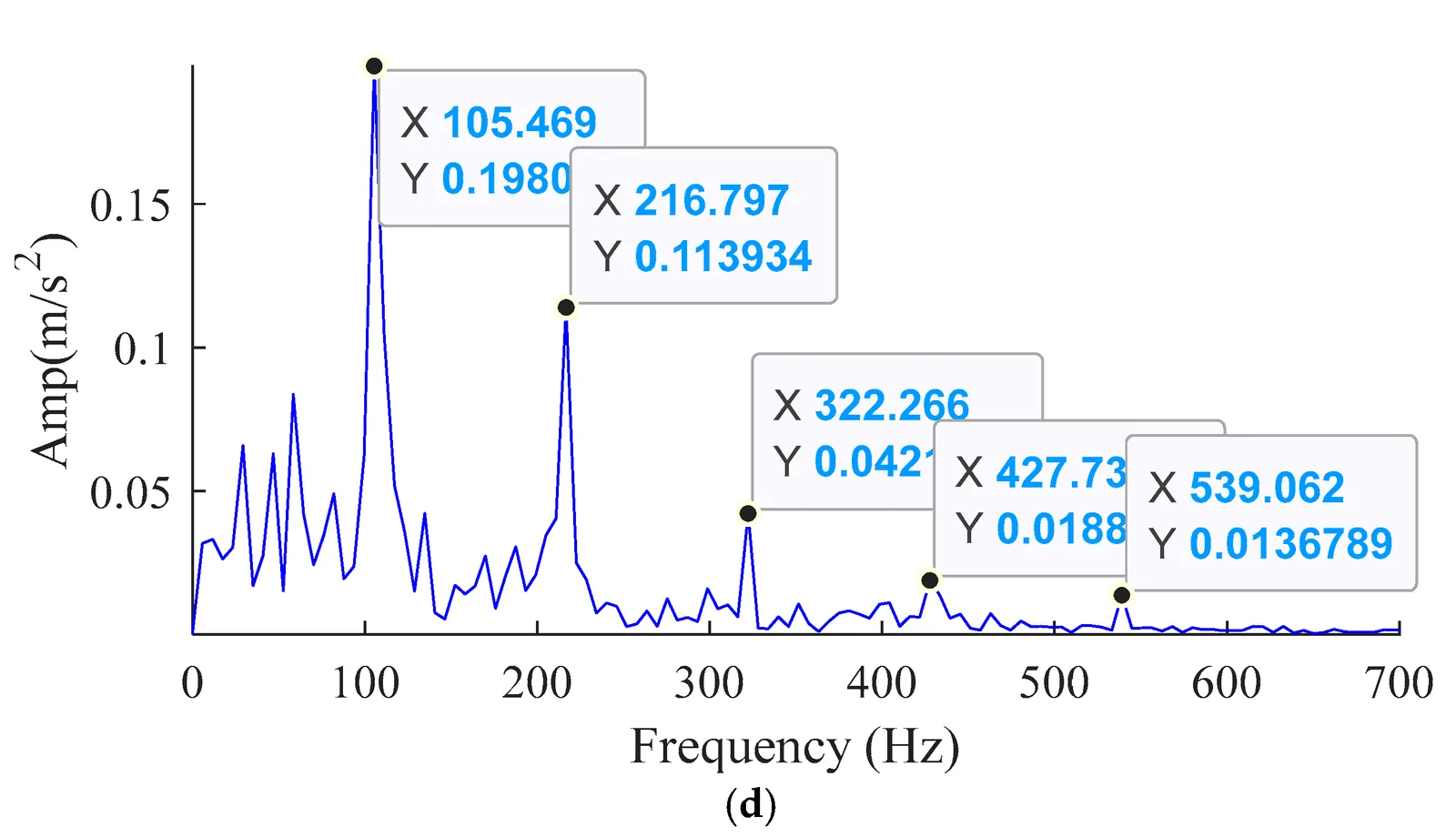
Comparative analysis of feature extraction methods for the outer ring: (**a**) VMD-Hilbert envelope spectrum of the optimal IMF component selected by kurtosis; (**b**) SVD-VMD-Hilbert envelope spectrum of the optimal IMF component; (**c**) DBO-VMD-Hilbert envelope spectrum of the optimal IMF component; (**d**) envelope spectrum of the optimal IMF2 component using the proposed SVD-DBO-VMD method.

To compare the spectral analysis effect of the sensitive features obtained by this method, a comparison is made with the VMD-Hilbert, SVD-VMD-Hilbert and DBO-VMD-Hilbert methods. The spectral analysis is performed on the IMF2 sensitive feature selected by the evaluation indicators through both feature extraction methods, and the effects are shown in Figure 10.

From the comparative analysis chart of the four feature extraction methods in Figure 10: In Figure 10a, the traditional VMD-Hilbert envelope analysis extracts a fault frequency of 161.1 Hz, which deviates from the theoretical outer race fault frequency of 104.57 Hz. The fault characteristic frequency is not distinctly visible in the broadband spectrum due to the presence of strong background interference and structural resonance components, which affects the diagnostic accuracy. Figure 10b presents the envelope spectrum of the SVD-VMD method, in which VMD is performed with default parameters following SVD-based denoising. The fault characteristic frequencies are accurately identified at the fundamental 1 f(105.5 Hz) and 2 f(216.8 Hz) components. Compared with the VMD-Hilbert approach, the background interference in the envelope spectrum is reduced, and the fault characteristic frequency components are more prominent. The SVD preprocessing effectively attenuates high-frequency noise while preserving the low-frequency fault information, thereby improving the clarity of the envelope spectrum. Nevertheless, third- and higher-order harmonics remain indiscernible, indicating that even after SVD preprocessing, the unoptimized VMD parameters preclude optimal separation of fault-related modes from residual signal components. In Figure 10c, the DBO-VMD method, which obtains the optimal parameter combination, decomposes the component to IMF3, and can effectively extract the fault frequencies of the outer ring 1 f(105.5 Hz), 2 f(216.8 Hz), and 3 f(322.3 Hz). Compared to the first three methods, the method presented in this paper provides better extraction of the fault characteristic frequency of the outer ring in Figure 10d. The SVD-DBO-VMD method effectively highlights the signal’s periodic pulses, enabling the Hilbert envelope spectrum to more accurately extract the fault frequency of the outer ring. The figure clearly shows the fault frequencies of the outer ring 1 f(105.5 Hz), 2 f(216.8 Hz), 3 f(322.3 Hz), 4 f(427.7 Hz), and 5 f(539.1 Hz). The periodic impact components of the bearing outer ring fault can be effectively extracted, which allows us to determine that a fault has occurred in the outer ring of the rolling bearing.

### 4.2. PU Data Experiment Analysis

**Figure 11:**
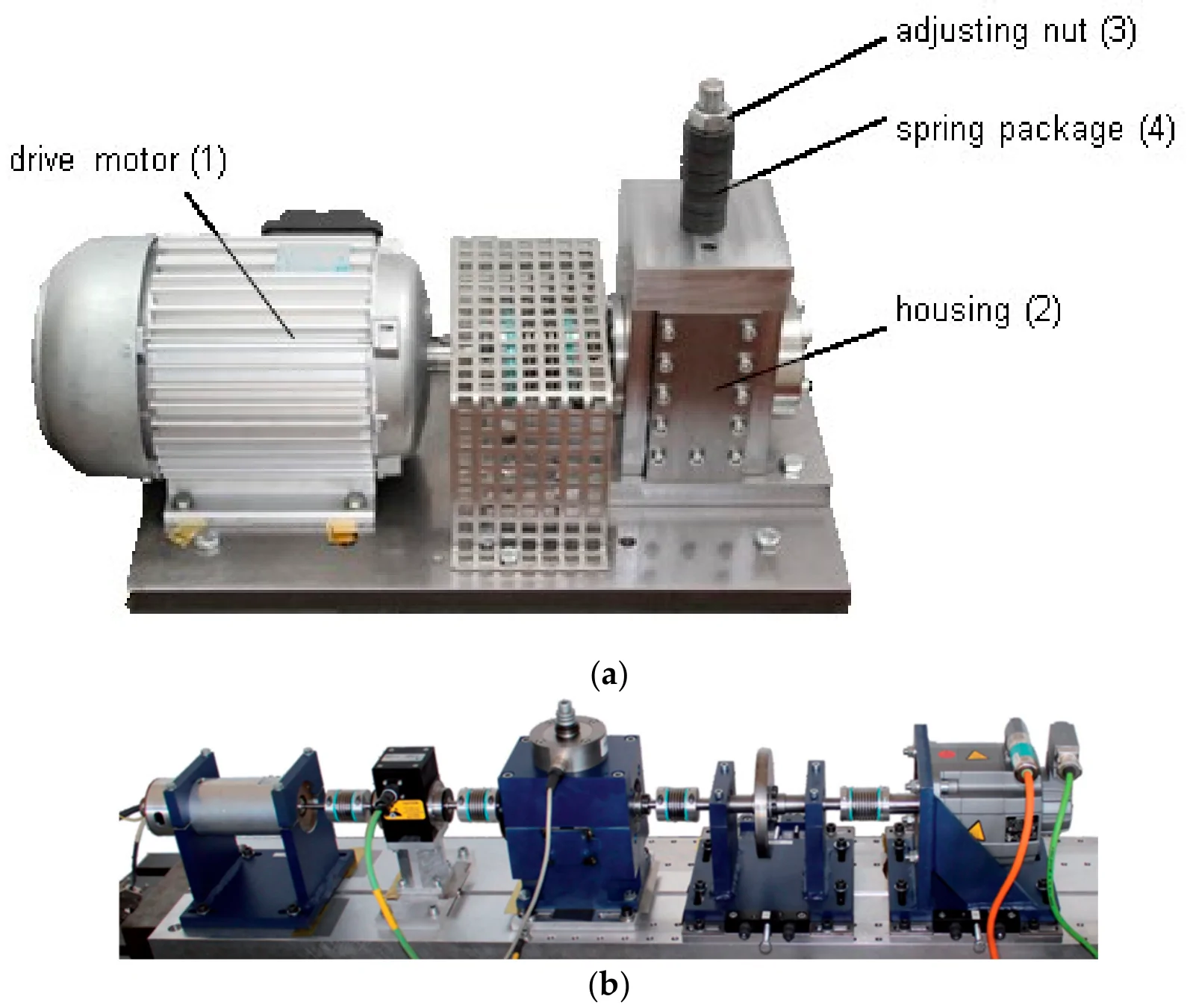
Experimental setups for the PU dataset: (**a**) Accelerated life test rig consisting of a three-phase induction motor, a spring-screw radial loading mechanism, and a low-viscosity oil lubrication system; (**b**) data acquisition test rig with a servo-driven shaft, four 6203 deep groove ball bearings mounted on the same shaft, vibration accelerometers mounted on the bearing housings, and a data acquisition system sampling at 64 kHz.

**Figure 12:**
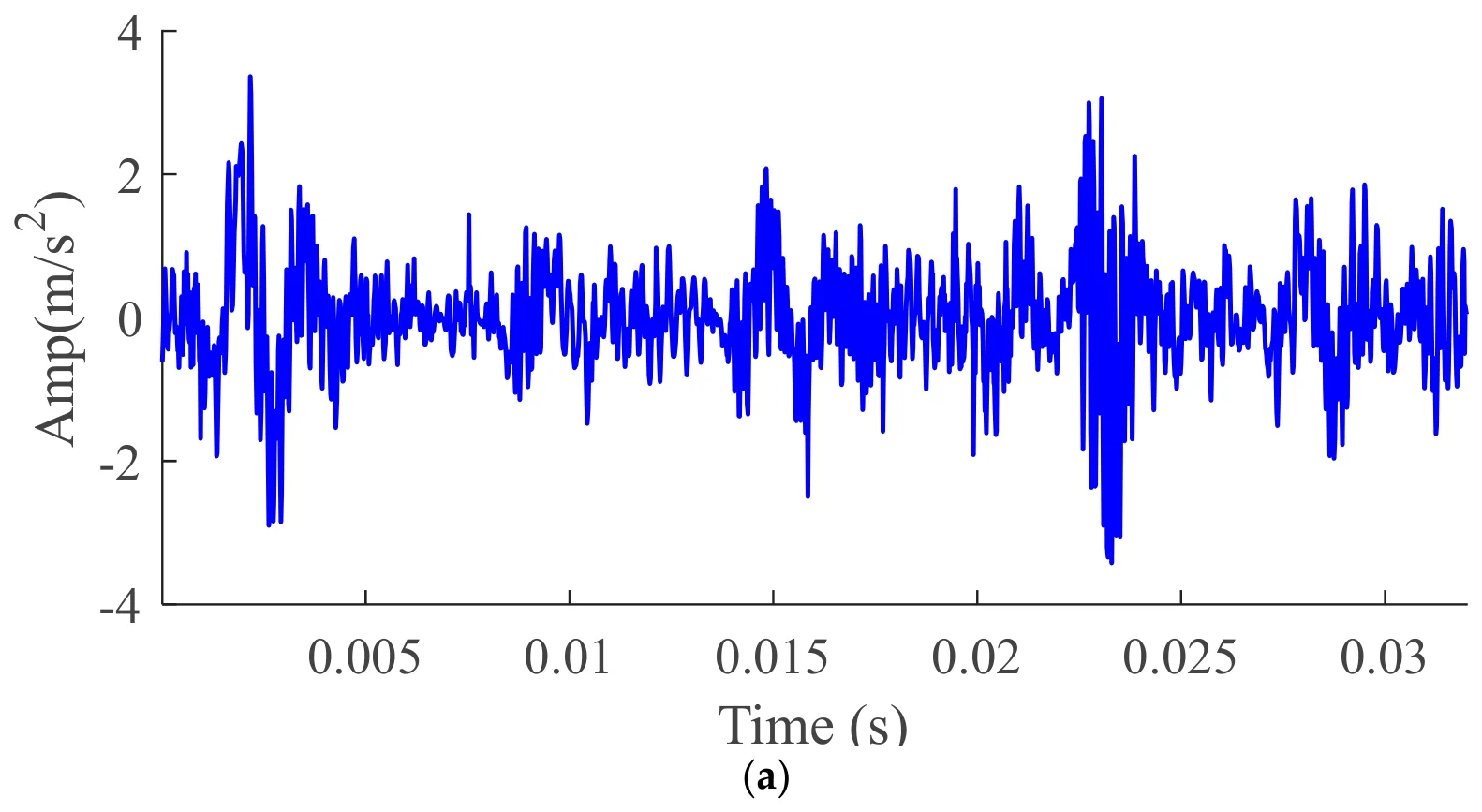

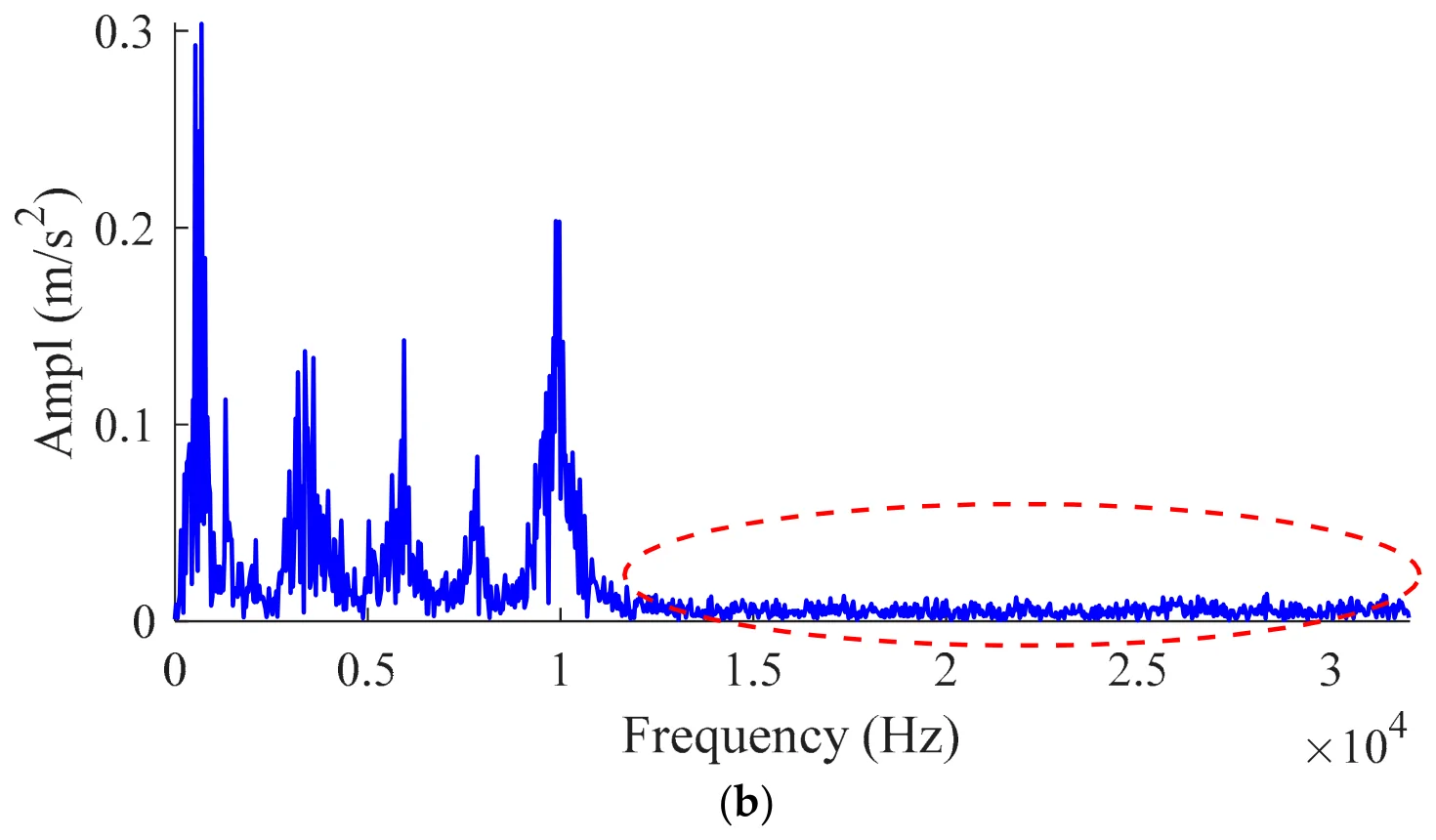
Inner ring waveform diagram: (**a**) Inner ring time domain waveform diagram; (**b**) inner ring frequency domain waveform diagram.

The formation of KB23 bearing data involves two stages. First, it was mounted on the PU accelerated life test rig (Figure 11a), where a spring-screw mechanism applied radial load to the bearing under aggressive conditions (high radial force and low-viscosity oil). After a few days, natural fatigue pitting developed into an inner-ring single-point fault (damage level 2). Then, the damaged bearing was transferred to the data acquisition test rig (Figure 11b), where four 6203 bearings are mounted on the same shaft. The test bearing is a 6203 deep groove ball bearing with the following specifications: inner diameter 17 mm, outer diameter 40 mm, thickness 12 mm, ball diameter 6.35 mm, and pitch diameter 28.5 mm. The shaft is driven by a servo motor with precise speed control, and vibration signals are acquired using accelerometers mounted on the bearing housings. The shaft rotational speed is 1500 rpm (25 Hz), and the sampling frequency is 64 kHz. The theoretical bearing defect frequencies are calculated as: inner race defect frequency (BPFI) = 156.25 Hz, outer race defect frequency (BPFO) = 103.36 Hz, and ball defect frequency (BSF) = 67.18 Hz. This paper uses these data from KB23 for experimental analysis. Its inner ring time-frequency domain waveform is shown in Figure 12. Compared with the time-domain graph in Figure 12a, the spectrum graph in Figure 12b better reflects that the original signal contains noise, necessitating noise reduction treatment of the original data to improve signal quality.

**Figure 13:**
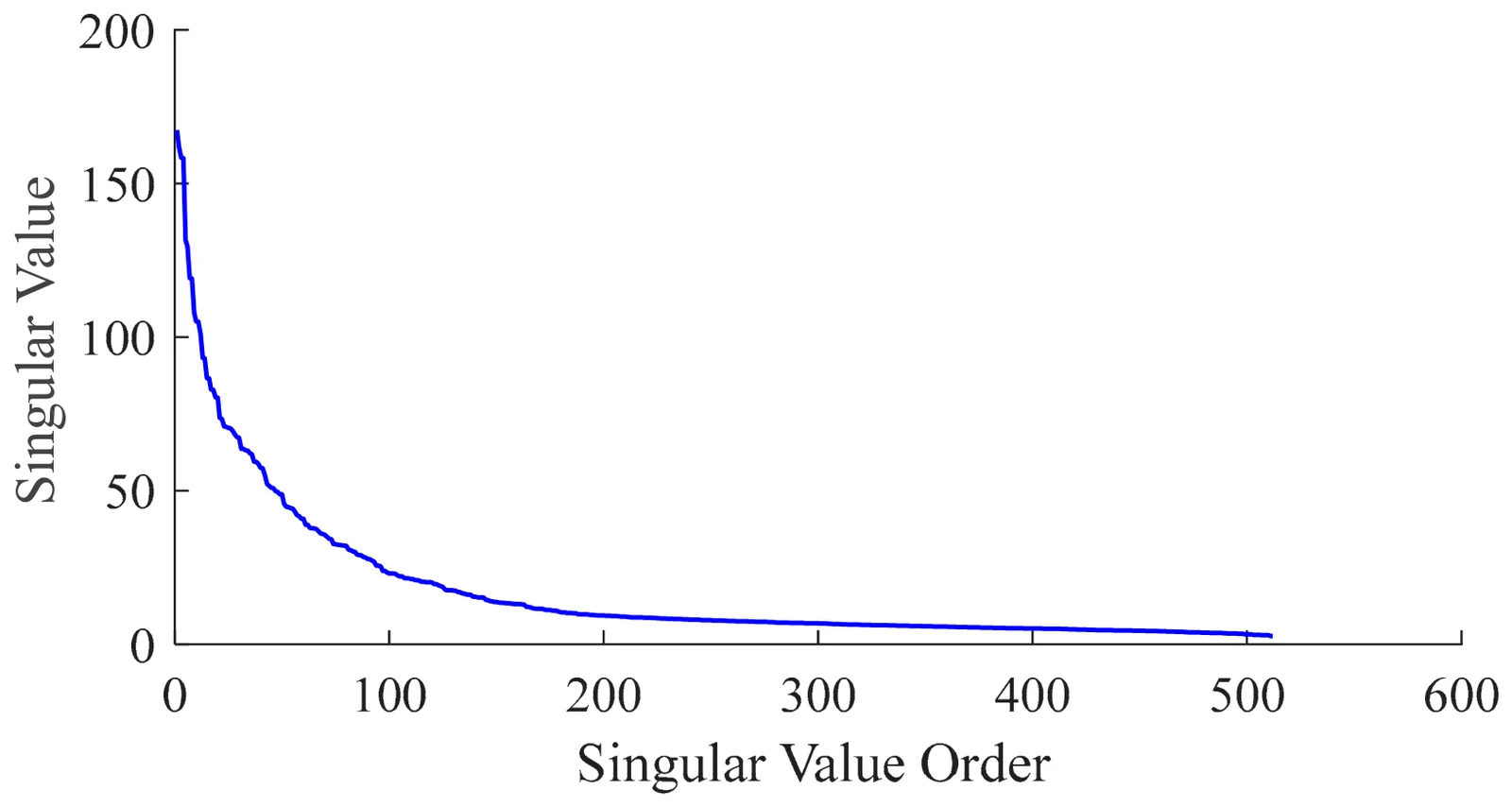
Distribution of singular values of faults in the inner ring.

**Figure 14:**
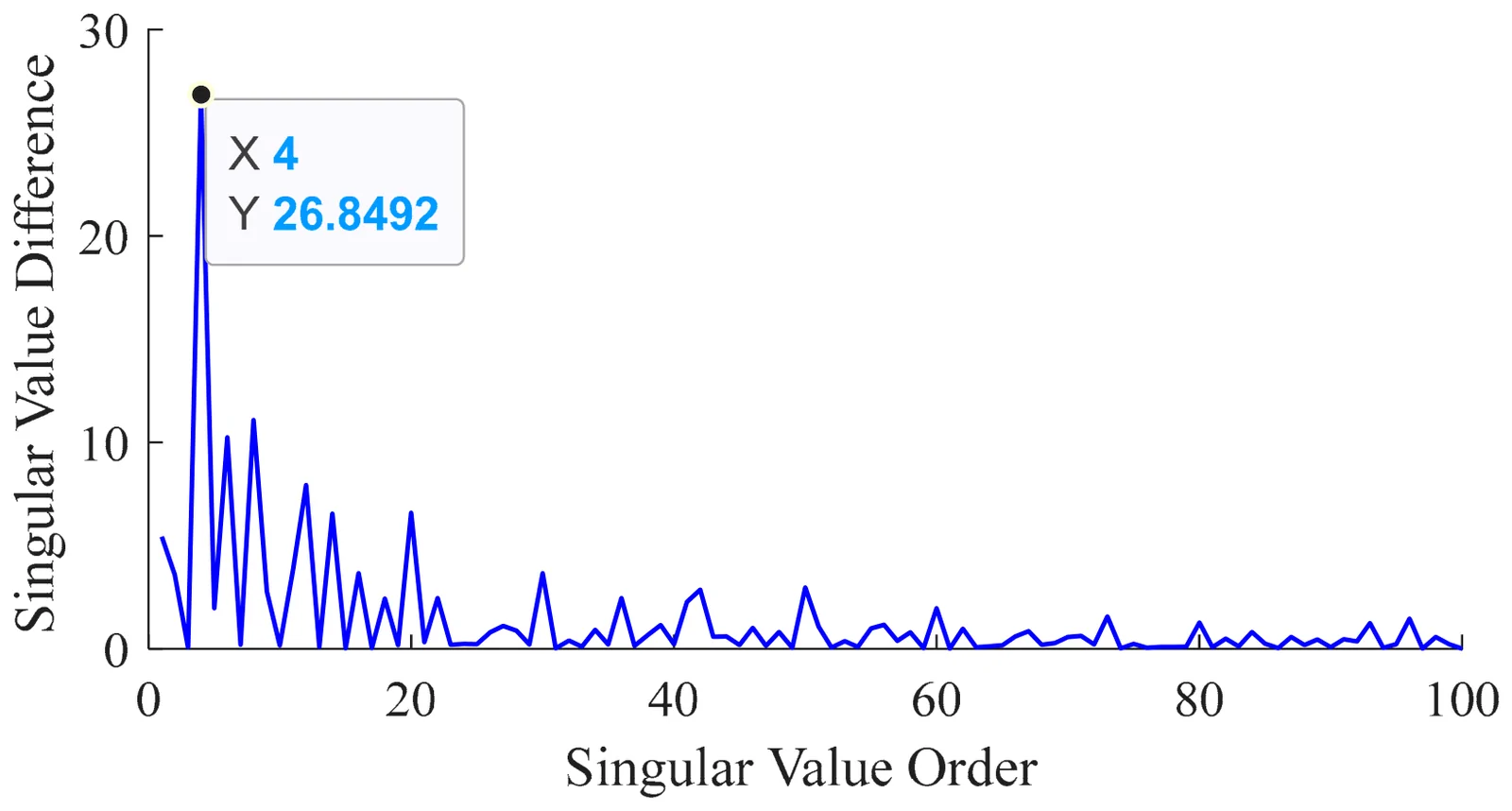
Inner ring fault singular value difference spectrum.

**Figure 15:**
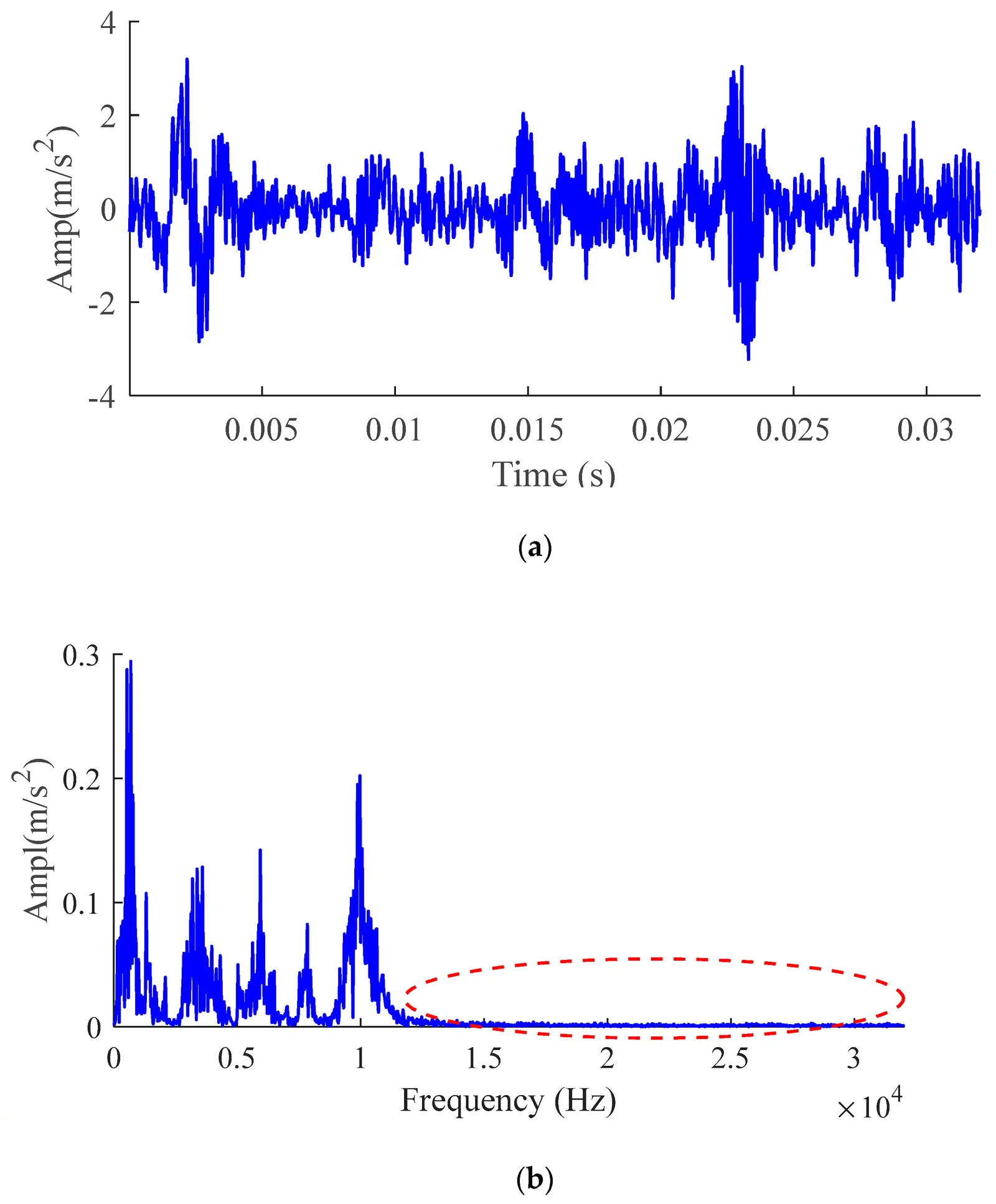
Time domain and frequency domain waveforms after SVD denoising: (**a**) Time waveform after SVD denoising; (**b**) frequency waveform after SVD denoising.

Using SVD for denoising preprocessing of the inner ring fault signal, the distribution of singular values and the singular value difference spectrum are shown in Figure 13 and Figure 14. After multiple experimental analyses, the denoising order indicated in the local part of the singular value difference spectrum in Figure 14 is selected for denoising. The denoising results of the time-domain and frequency-domain graphs of the inner ring fault are shown in Figure 15.

**Figure 16:**
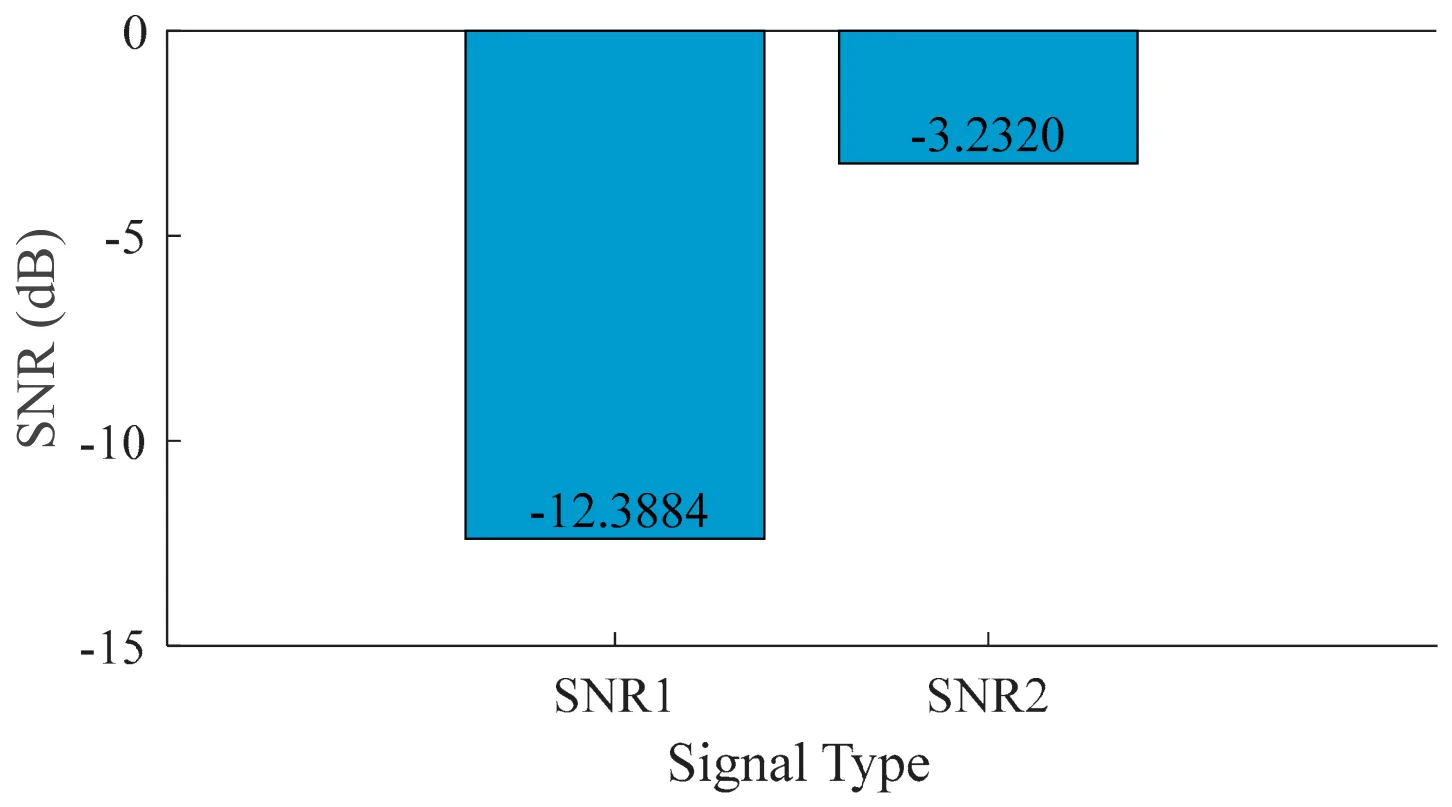
Comparison chart of SNR before and after noise reduction of the inner ring.

Comparing the denoised signal (Figure 15) with the undenoised signal (Figure 12), it can be observed that the noise has been effectively suppressed, irrelevant noise frequency bands have been removed, and the frequency bands of the useful signal have been preserved. To further verify the denoising effect, the signal-to-noise ratio (SNR) of the original signal (Figure 12, denoted as SNR1) and the SVD-denoised signal (Figure 15, denoted as SNR2) were calculated respectively via spectral estimation following the procedure described in Section 2.2, as shown in Figure 16.

From Figure 16, it can be seen that SNR1 = −12.3884 dB and SNR2 = −3.2320 dB. SNR2 is approximately 9.15 dB higher than SNR1, indicating that the signal quality is significantly better than that of SNR1. The signal has lower noise interference and higher clarity, achieving the expected noise reduction effect.

**Figure 17:**
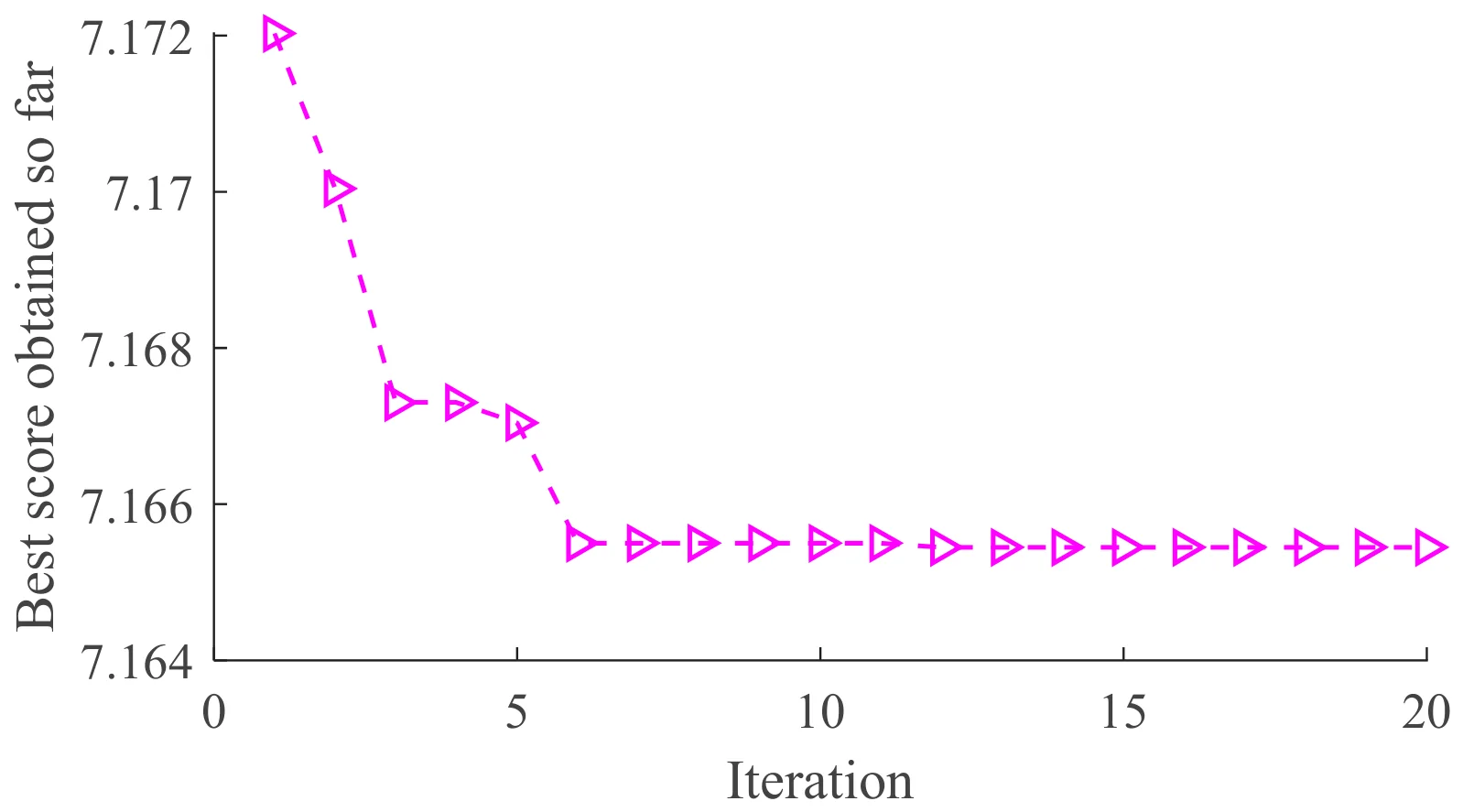
Iterative plot of VMD parameters for DBO of inner ring data.

**Figure 18:**
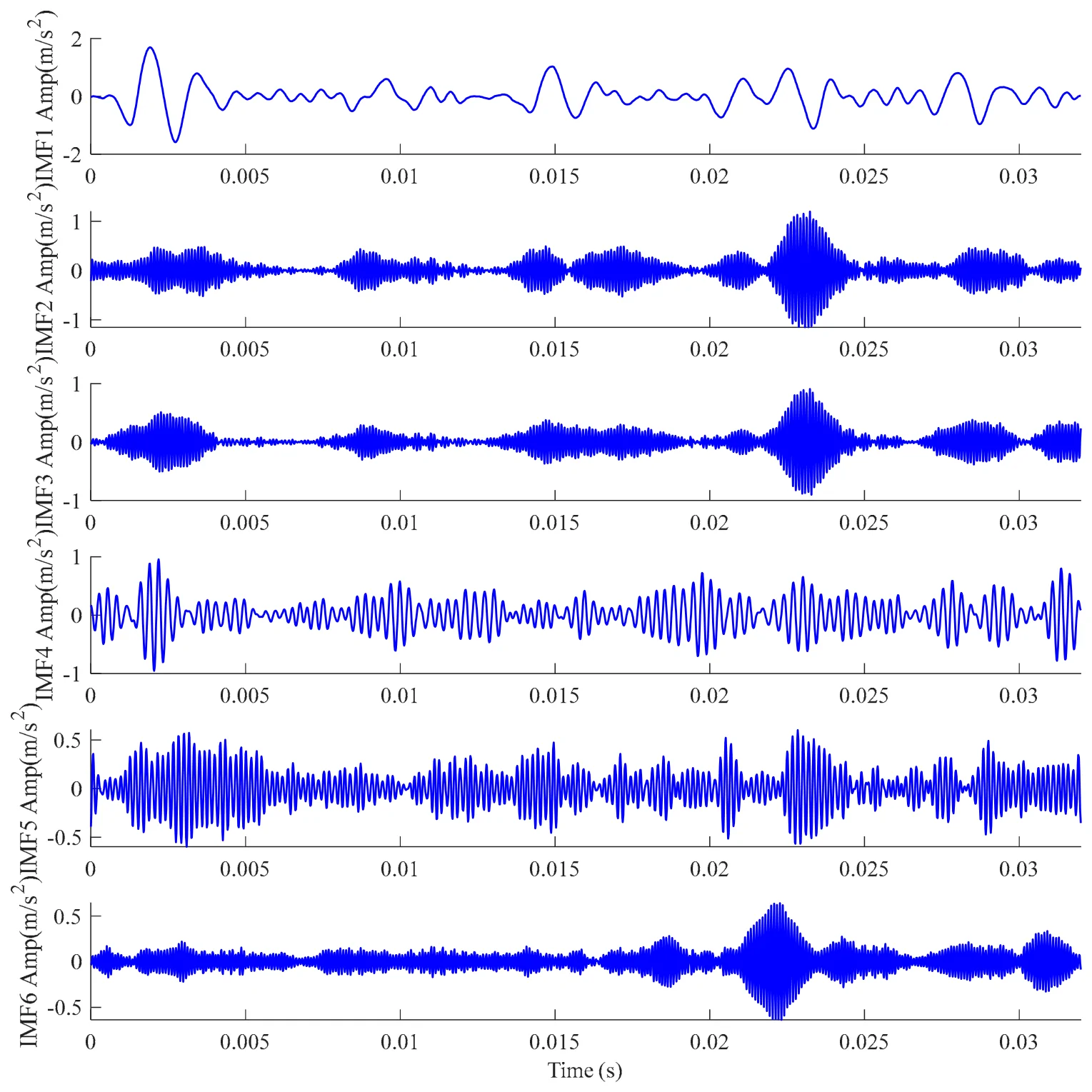
VMD of inner ring fault signal after SVD noise reduction.

The parameter settings for DBO-optimized VMD are the same as before. From Figure 17, it can be observed that when the number of iterations reaches 7, the fitness function quickly converges to a stable state, with the optimal fitness function value being 7.1654, and the best parameter combination for VMD is K=6 and α=184. The pitting fault signal of the inner ring, after being decomposed by VMD with the set parameters, results in 6 IMF components as shown in Figure 18.

**Figure 19:**
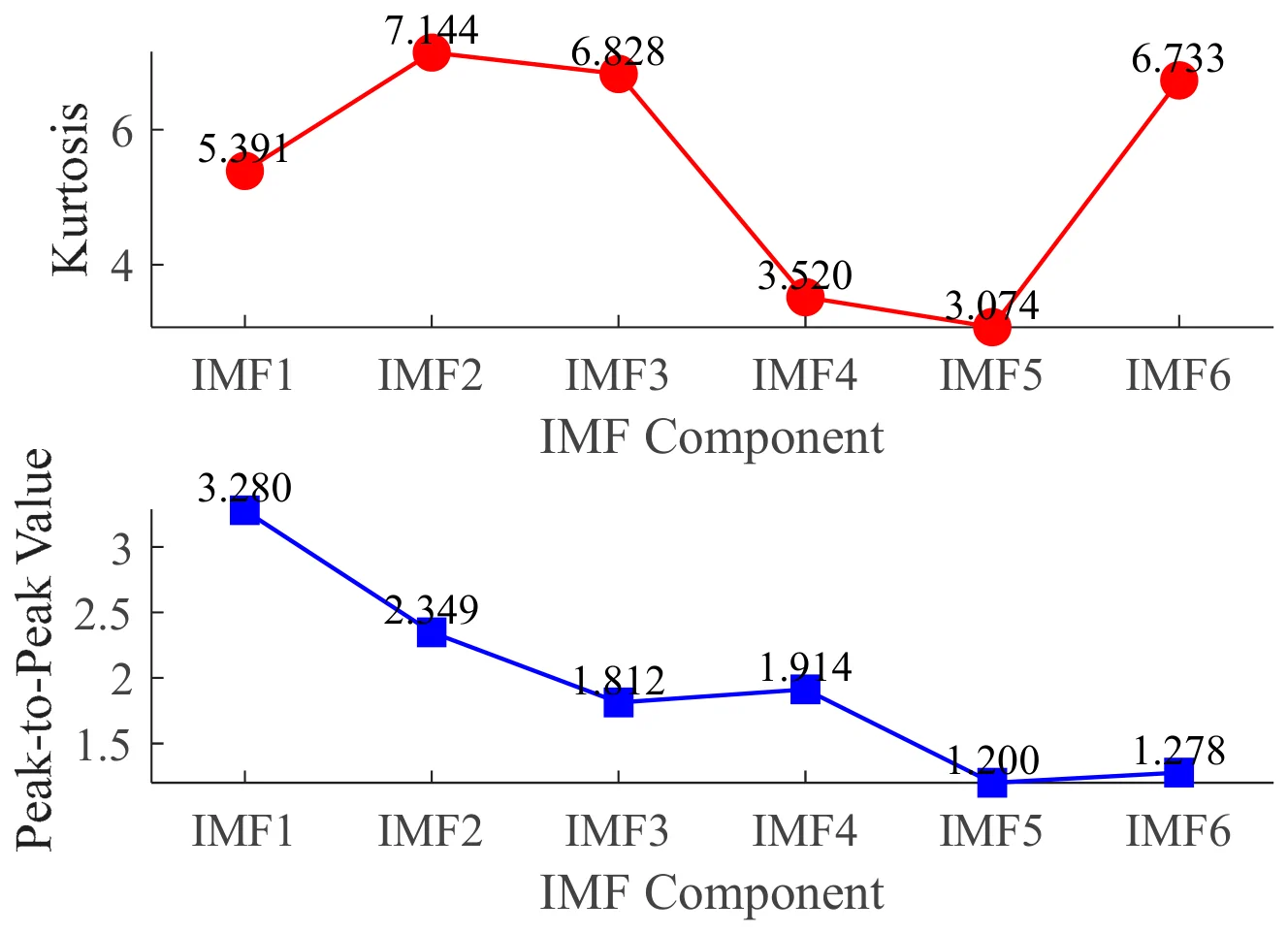
Graph of two evaluation indicators for each IMF component in the inner ring.

From the components obtained by VMD, we select an IMF that contains the most fault information as the sensitive component, and Figure 19 shows the two evaluation indicator graphs for each IMF component of the inner ring fault.

The combined kurtosis-peak-to-peak ratio metric prioritizes kurtosis while using peak-to-peak as a secondary measure to mitigate biases caused by energy decay. As shown in Figure 19, IMF2 has the highest kurtosis, significantly higher than the other components, indicating that it contains the most significant impact components and is suitable for extracting the fault frequency. Although IMF1 has the highest peak-to-peak ratio, its kurtosis is low, suggesting that it may contain a significant amount of noise or non-fault impacts. Therefore, IMF2 is selected as the sensitive component.

**Figure 20:**
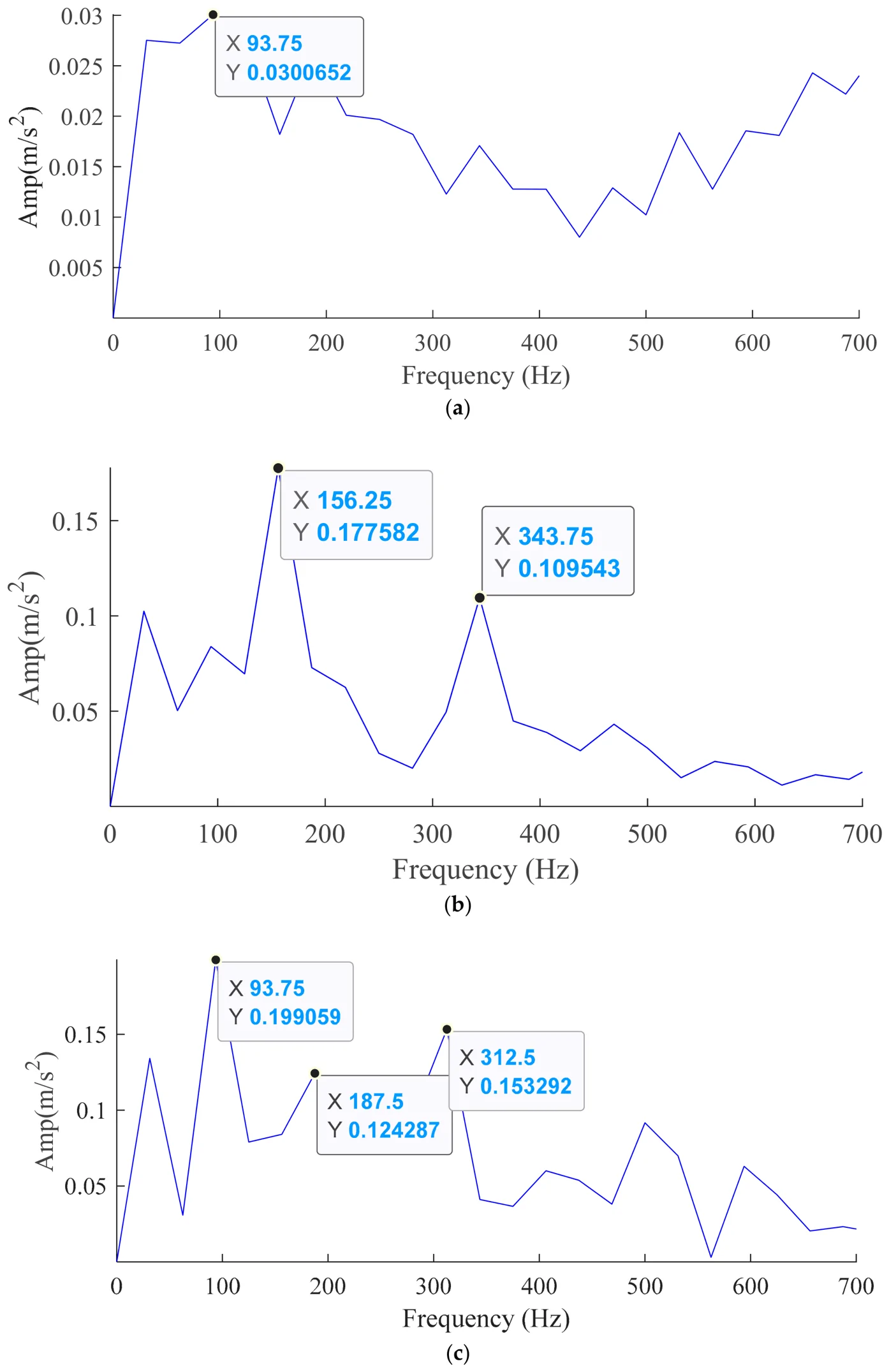

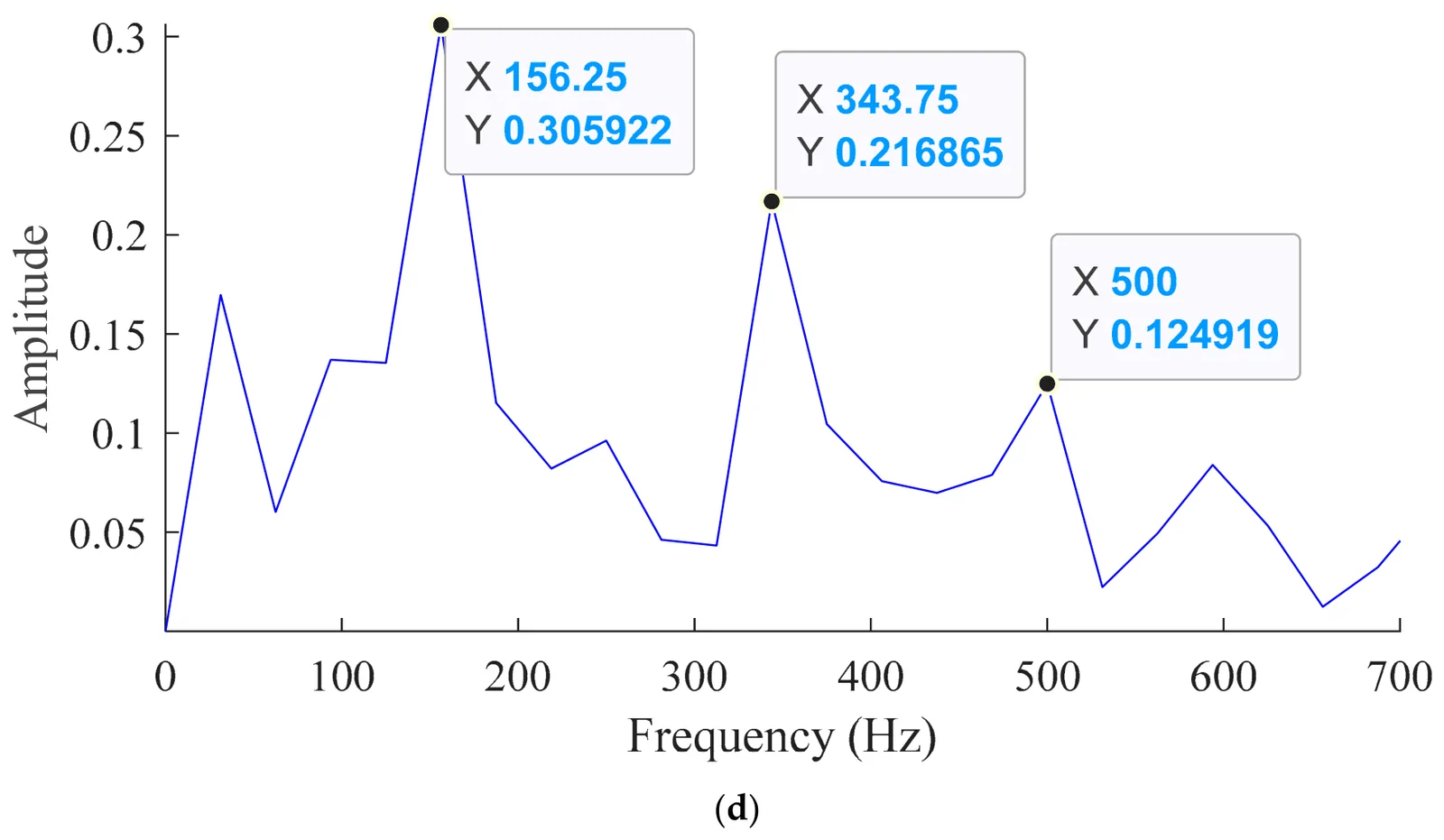
Comparative analysis of feature extraction methods for the outer ring: (**a**) VMD-Hilbert envelope spectrum of the optimal IMF component selected by kurtosis; (**b**) SVD-VMD-Hilbert envelope spectrum of the optimal IMF component; (**c**) DBO-VMD-Hilbert envelope spectrum of the optimal IMF component; (**d**) envelope spectrum of the optimal IMF component using the proposed SVD-DBO-VMD method.

To compare the spectral analysis effect of the sensitive features obtained by this method with traditional methods, a comparison is made with the VMD-Hilbert, SVD-VMD-Hilbert and DBO-VMD-Hilbert methods. The spectral analysis is performed on the IMF2 sensitive feature selected by the evaluation indicators through both feature extraction methods, and the effects are shown in Figure 20.

Figure 20 shows the comparative analysis charts of the four feature extraction methods. In Figure 20a, the traditional VMD-Hilbert method envelope analysis extracted a fault frequency of 93.75 Hz, which fails to accurately extract the multiple frequencies of the inner ring fault frequency. Figure 20b presents the envelope spectrum obtained by applying VMD with default parameters following SVD-based denoising. The fault characteristic frequencies are correctly identified at the dominant peak 1 f(156.25 Hz) and 2 f(343.75 Hz), corroborating the periodic nature of the inner race defect. However, the third harmonic remains indiscernible, with a comparatively elevated noise floor persisting between the harmonic components. Notably, the second harmonic 2 f(343.75 Hz) exhibits a frequency offset of 31.25 Hz relative to the theoretical fault frequency of 312.5 Hz. These observations indicate that although SVD denoising effectively suppresses random noise, fixed VMD parameters nevertheless compromise the precision of harmonic localization. The omission of DBO precludes adaptive adjustment of the mode number and penalty factor to the specific signal characteristics, resulting in incomplete harmonic extraction and residual mode aliasing. Figure 20c presents the envelope spectrum of the DBO-VMD method without SVD denoising. The dominant peak appears at 93.75 Hz, corresponding to the outer race defect frequency rather than the inner race defect frequency. This indicates that DBO alone is insufficient to rectify fundamental misclassification induced by strong background noise. The coexistence of outer race defect 2 f(187.5 Hz) and the inner race defect 2 f(312.5 Hz) demonstrates severe mode aliasing arising from noise interference. These findings confirm that although DBO effectively optimizes VMD parameters, the omission of SVD preprocessing results in erroneous mode separation and fault misclassification attributable to strong background noise. Compared to the first three methods, the method presented in this paper, as shown in Figure 20d, provides better extraction of the bearing fault characteristic frequencies, effectively highlighting the signal’s periodic pulses and enabling the Hilbert envelope spectrum to more accurately extract the fault frequency. The figure clearly displays prominent peaks at 1 f(156.25 Hz), 2 f(343.75 Hz), and 3 f(500 Hz). The fundamental component at 156.25 Hz exactly matches the theoretical BPFI, confirming the inner race fault. The peak at 343.75 Hz is identified as a modulation sideband of the second harmonic (2 × BPFI = 312.5 Hz); this offset is attributed to the modulation effect between the BPFI harmonics and the shaft rotational frequency ($f_{r}$= 25 Hz), combined with possible speed fluctuation and measurement uncertainty. The third peak at 500 Hz corresponds to a 3 × BPFI sideband. The presence of the exact fundamental BPFI, together with the clearly identifiable harmonic family and modulation sidebands, provides sufficient evidence for the inner race fault diagnosis.

## 5. Conclusions

In response to the challenge of poor quality in fault information extraction for rolling bearings, this paper proposes a novel hybrid SVD-DBO-VMD method for enhanced fault feature extraction. The main contributions and findings are summarized as follows:

(1) The proposed method establishes an effective framework for rolling bearing fault feature extraction, integrating adaptive SVD denoising, intelligent DBO parameter optimization, and multi-indicator IMF selection. The singular value difference spectrum provides a robust criterion for determining the SVD denoising order, while the DBO algorithm with minimum envelope entropy as the fitness function successfully optimizes the VMD parameters. The kurtosis-crest factor joint criterion effectively selects the IMF component richest in fault information.

(2) Experimental validation on CWRU and PU datasets confirms that the proposed method outperforms conventional VMD-Hilbert, SVD-VMD-Hilbert, and DBO-VMD-Hilbert approaches in terms of harmonic extraction capability and fault identification accuracy. The method successfully extracts up to five harmonic components of the fault frequency, enabling more reliable fault type identification.

(3) While the experimental results successfully demonstrate the effectiveness of the proposed hybrid method, several limitations of the current study should be acknowledged. First, the computational cost of the SVD-DBO-VMD method is higher than that of the traditional VMD-Hilbert approach due to the iterative optimization process. Second, the performance of the DBO algorithm is sensitive to its hyperparameters, such as population size and number of iterations, which can affect real-time fault diagnosis capability if not properly tuned. Third, the current framework relies solely on handcrafted features (kurtosis and peak-to-peak value) for IMF selection; deeper integration with deep-learning-based feature extraction has not been explored. Future work will focus on developing lightweight optimization strategies to reduce computational overhead, systematically investigating the sensitivity of DBO parameters, and fusing the proposed method with deep learning architectures to further enhance fault diagnosis accuracy and robustness.

## Figures and Tables

**Table 1:** SKF6205 bearing parameters.

Inside Diameter (in)	Outside Diameter (in)	Thickness (in)	Ball Diameter (in)	Pitch Diameter (in)
0.9843	2.0472	0.5906	0.3126	1.537

**Table 2:** DBO parameter setting.

Parameters	Value	Description
ub	[100 3]	Upper search limit of VMD parameters [α_max, K_max]
lb	[2500 10]	Lower search limit of VMD parameters [α_min, K_min]
dim	2	Number of optimization variables (α and K)
Max_iter	20	Maximum number of DBO iterations
SearchAgents_no.	25	Number of dung beetles in the population

## Data Availability

Data available in a publicly accessible repository.
